# Novel Antifungal Pyridine Alkaloids from Endophytic Fungus *Penicillium citrinum* VDL118

**DOI:** 10.3390/jof12040296

**Published:** 2026-04-20

**Authors:** Mei Yang, Shan Hu, Zhi-Yu Zhang, Fa-Zhong Yang, Xiao-Qin Yang, Si-Da Xie, Ying-Jun Zhang, Ping Zhao, Guo-Lei Zhu

**Affiliations:** 1 Key Laboratory of State Forestry and Grassland Administration on Highly-Efficient Utilization of Forestry Biomass Resources in Southwest China, Southwest Forestry University, Kunming 650233, China; 17787982850@163.com (M.Y.); hushan@163.com (S.H.); zzy-529_654@swfu.edu.cn (Z.-Y.Z.); yangfazhong105@163.com (F.-Z.Y.); yangxiaoqin@swfu.edu.cn (X.-Q.Y.); dream102035@163.com (S.-D.X.); 2 Key Laboratory of Phytochemistry and Natural Medicines, Kunming Institute of Botany, Chinese Academy of Sciences, Kunming 650201, China; zhangyj@mail.kib.ac.cn

**Keywords:** *Vaccinium dunalianum*, endophytic fungi, *Penicillium citrinum*, pyridine alkaloids, antifungal activity

## Abstract

Three novel alkaloids, penicitrioids A–C (**1**–**3**), and two known compounds (**4**–**5**) were isolated from the ethyl acetate (EtOAc) extract of the solid fermentation of *Penicillium citrinum* VDL118, an endophytic fungus harbored in the leaves of *Vaccinium dunalianum* Wight (Ericaceae), a perennial evergreen shrub native to the southwestern regions of China, Myanmar, and Vietnam. Compounds **1** and **2** are novel pyridine alkaloids characterized by an unprecedented dihydrofuro[3,4-c]pyridine core, while **3** features a distinct pyrrolo[3,4-c]pyridine framework. Their structures were unambiguously established by comprehensive spectroscopic analysis and electronic circular dichroism (ECD) calculations. In vitro antifungal assays revealed that compounds **1**–**5** exhibited moderate to potent inhibitory effects against five tested phytopathogenic fungi, with minimum inhibitory concentrations (MICs) ranging from 3.1 to 100 μg/mL. Notably, four of them (**1**–**4**) displayed broad-spectrum and potent activity against *Gloeophyllum trabeum*, *Coriolus versicolor*, *Fusarium solani*, and *Botrytis cinerea*, with MIC values as low as 3.1–12.5 μg/mL. Furthermore, a plausible biosynthetic pathway for compounds **1**–**3** was proposed.

## 1. Introduction

Plant diseases pose a significant threat to global food security and sustainable agricultural development. Although chemical fungicides are widely used for disease management, their extensive and often indiscriminate use has led to severe drawbacks, including the emergence of resistant pathogen populations and escalating concerns regarding agro-product safety [1,2,3,4]. Consequently, the development of novel, eco-friendly biopesticides with high efficacy and low toxicity is an urgent priority. Endophytic fungi, which establish mutualistic symbioses with host plants by colonizing their internal tissues without causing plant disease, have emerged as a prolific reservoir of bioactive secondary metabolites for biocontrol applications [5,6,7].

*Vaccinium dunalianum* Wight (Ericaceae), an evergreen shrub, is a particularly valuable source of both traditional medicine and unique endophytic biodiversity. Its dried leaf buds, known locally as “Sparrow Mouth Tea”, have been used ethnopharmacologically by the Yi people since the Ming Dynasty [8,9]. Beyond its documented high content of caffeoyl arbutin derivatives [10], recent investigations have highlighted *V. dunalianum* as a rich host for endophytic fungi capable of producing structurally diverse and biologically active natural products [11]. As part of our ongoing initiative to discover novel antifungal natural products, we systematically screened extract libraries derived from diverse endophytic fungi isolated from this plant. This effort culminated in the isolation of a series of structurally unique natural compounds exhibiting potent activity against agriculturally significant phytopathogens [12,13,14,15,16]. Building upon this foundation, *Penicillium citrinum* VDL118, a dominant endophytic fungus also isolated from *V. dunalianum*, was selected as the subject of this study due to its potent antimicrobial secondary metabolites [17,18,19].

**Figure 1:**
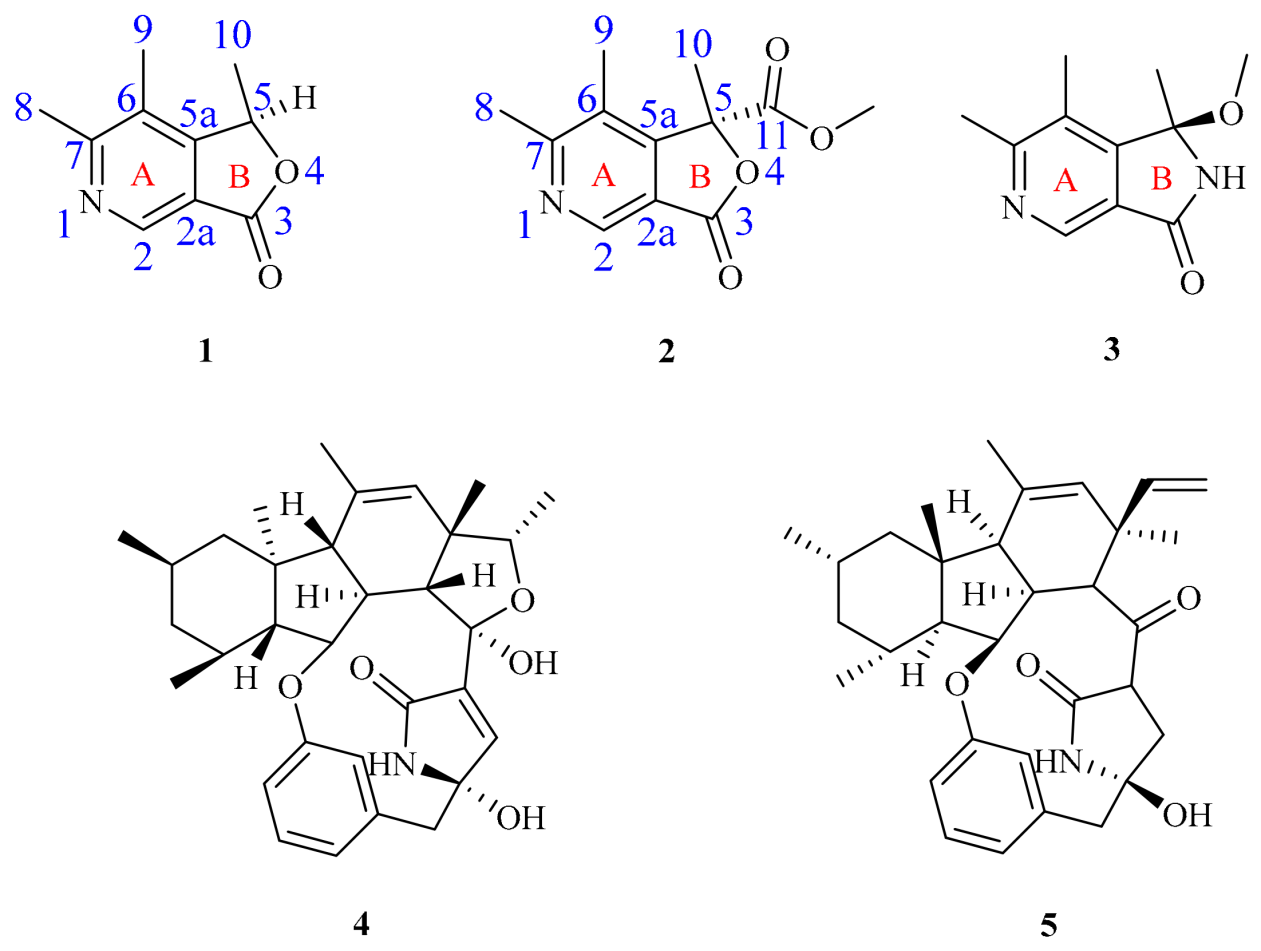
Structures of compounds **1**–**5**.

*Penicillium citrinum* serves as a key industrial strain not only for antibiotic production but also for the synthesis of various enzymatic preparations, including glucosidases, cellulases, and nucleases [20]. This fungus exhibits remarkable metabolic diversity. Since the initial discovery of mevastatin from *P. citrinum* in the 1970s, subsequent studies have identified a wide array of bioactive compounds—including polyketides, sterols, terpenoids, and alkaloids—from strains isolated from diverse habitats, underscoring its substantial potential for secondary metabolism [21,22,23]. Among microbial metabolites, alkaloids represent a class of nitrogen-containing heterocyclic compounds primarily recognized from plants [24], while also constituting an essential component of fungal secondary metabolites [25]. Owing to their frequent structural novelty and significant biological activities—such as antimicrobial, antitumor, and anti-inflammatory effects [26,27,28]—alkaloids remain a major focus in natural product-based drug discovery. Among the various alkaloid families, pyridine alkaloids have consistently emerged as a frontier hotspot in natural product research due to their unique nitrogen-containing heterocyclic skeletons and diverse biological activities. In recent years, a considerable number of structurally novel pyridine alkaloids have been discovered from *Penicillium* fungi inhabiting diverse ecological niches, clearly demonstrating the considerable potential of this microbial genus in chemical ecology and drug lead discovery. For instance, Gan et al. isolated penaloidines A and B from the plant endophytic fungus *Penicillium* sp. KYJ-6, which possess an unprecedented tetrahydrofuranonaphthyridine skeleton and exhibit significant acetylcholinesterase inhibitory activity [29]. Marine-derived fungi have also shown remarkable chemical diversity. From the deep-sea-derived *P*. *citrinum* SCSIO DF147, researchers isolated a series of pyrroline-derived alkaloids, among which compound MOPE exhibited 100% larvicidal activity against *Plutella xylostella*, suggesting its potential agricultural application value [30]. Furthermore, pyridone alkaloids such as penicipyridones A–K, discovered from the marine-derived *Penicillium oxalicum* QDU1, have garnered widespread attention due to their various bioactivities, including anti-inflammatory and cytotoxic effects [31]. Significant progress has also been made in the area of antifungal activity. Shiraishi et al. identified secondary metabolites with significant inhibitory activity against two important plant pathogenic fungi—*Fusarium oxysporum* and *Sclerotinia sclerotiorum*—from the fermentation extract of *P*. *citrinum* isolated from *Anticarsia gemmatalis* larvae. The chloroform crude extract exhibited inhibition rates of 86.9% and 100% against these two fungi, respectively, demonstrating application potential in agricultural disease control [32]. In another study, Rukachaisirikul et al. isolated the known benzopyranone compounds coniochaetones A and C from the marine-derived *P*. *citrinum* PSU-MF100, which exhibited moderate antifungal activity against *Candida albicans* and *Cryptococcus neoformans*, with MIC values of 16 μg/mL and 64 μg/mL, respectively, indicating the potential medicinal value of these compounds against human fungal infections [33]. In summary, *Penicillium* fungi, particularly strains originating from specialized ecological niches such as plant endophytes and marine environments, represent not only important sources for discovering structurally novel pyridine alkaloids but also valuable resources for identifying bioactive metabolites with agricultural and pharmaceutical potential. Against this background, five alkaloids (**1**–**5**; Figure 1)—including three new compounds, penicitrioids A–C (**1**–**3**), and two known analogs (**4**–**5**)—were obtained from the EtOAc extract of the solid fermentation cultures of *P. citrinum* VDL118, an endophytic fungus derived from *V. dunalianum*. The planar structures of all compounds were elucidated using high-resolution mass spectrometry (HRMS) coupled with one- and two-dimensional nuclear magnetic resonance (NMR) spectroscopy. Additionally, the antifungal activities of all isolates against five pathogenic fungi were evaluated, with MICs ranging from 3.1 to 100 μg/mL.

## 2. Experimental Section

### 2.1. General Experimental Procedures

Proton (^1^H) and carbon-13 (^13^C) NMR spectra were recorded on a Bruker DRX-500 spectrometer at room temperature (manufactured by Bruker, headquartered in Karlsruhe, Germany), using tetramethylsilane (TMS) as an internal standard. Coupling constants (*J*) were reported in hertz (Hz), and chemical shifts were expressed in *δ* (ppm). HRMS data were acquired on an Agilent 6200 Q-TOF mass spectrometer equipped with an electrospray ionization (ESI) source (manufactured by Agilent, headquartered in Santa Clara, California, USA). Ultraviolet (UV) spectra were recorded on a UV-2401 PC ultraviolet–visible (UV-VIS) spectrophotometer (manufactured by Shimadzu, headquartered in Kyoto, Japan). IR spectra were measured on a Bruker Tensor 27 Fourier-transform infrared (FTIR) spectrometer with samples prepared as potassium bromide (KBr) pellets (manufactured by Bruker, headquartered in Karlsruhe, Germany). Optical rotations were determined on an Autopol VI automatic polarimeter (manufactured by Rudolph Research Analytical, Hackettstown, NJ, USA). Circular dichroism (CD) spectra were recorded on a spectropolarimeter (manufactured by JASCO, Tokyo, Japan).

Semi-preparative high-performance liquid chromatography (HPLC) separations were performed on an Agilent 1260 system equipped with a reversed-phase (RP) C18 column (Zorbax SB, 9.4 mm × 250 mm, 5 μm; Kyoto, Japan). Column chromatography was carried out using various stationary phases: Sephadex LH-20 (20–150 μm, Pharmacia, Uppsala, Sweden), YMC*GEL ODS-A-HG (50 μm, YMC Group, Japan), macroporous resin D101 (Sichuan Zhongcheng, Chengdu, China), and silica gels of different mesh sizes (100–200, 200–300, and 300–400 mesh; Qingdao Haiyang Chemical Co., Ltd., Qingdao, China). Thin-layer chromatography (TLC) analyses were conducted on pre-coated silica gel GF_254_ plates (200–250 μm, Qingdao Haiyang Chemical Co., Ltd., Qingdao, China). Visualization of spots was achieved by spraying with a 10% H_2_SO_4_ solution in ethanol, followed by heating.

### 2.2. Fungal Material and Fermentation

The endophytic fungus *P. citrinum* VDL118 was isolated and identified from the leaves of *V. dunalianum* collected in Wuding County, Yunnan Province [10]. Through DNA extraction, amplification, purification, and sequencing, the internal transcribed spacer (ITS) sequence of this strain was obtained. The acquired ITS sequence was subsequently subjected to BLAST analysis (BLAST 2.17.0) at the National Center for Biotechnology Information (NCBI), revealing the highest homology with *P. citrinum*. The strain was thus identified as *P. citrinum*. Five plant pathogenic fungi (obtained from the China General Microbiological Culture Collection Center) were used in the study: *Gloeophyllum trabeum* (CFCC 86617), *Coriolus versicolor* (CFCC 5336), *Fusarium solani* (CGMCC 3.2889), *Botrytis cinerea* (CGMCC 3.3790), and *Fusarium graminearum* (CGMCC 3.4733). All strains were deposited in the Key Laboratory of Southwest Mountain Forest Resources Conservation and Utilization (Ministry of Education), Southwest Forestry University.

The *P*. *citrinum* strain VDL118, preserved at −80 °C, was revived on potato sucrose agar (PSA) medium. The PSA medium consists of potato infusion (200 g/L), sucrose (18 g/L), and agar (9 g/L) at natural pH. The activated strain was subsequently inoculated onto fresh PSA plates and incubated at 28 ± 1 °C for 7 days in a constant-temperature incubator, a temperature range consistent with the optimal growth conditions (28–30 °C) reported for *P. citrinum*. After incubation, mycelial plugs (5 mm in diameter) were aseptically excised using a sterile cork borer and transferred into 300 Erlenmeyer flasks (500 mL capacity each) for solid-state fermentation. Each flask contained 50 g of rice and 50 mL of distilled water, which had been soaked overnight and sterilized by autoclaving at 121 °C for 20 min. Rice is widely employed as a substrate in solid-state fermentation due to its abundant carbon sources and nutrients, which facilitate the accumulation of fungal secondary metabolites [20,21,22,23]. All flasks were incubated statically at 28 ± 1 °C for 30 days in a temperature-controlled culture room. Static cultivation promotes uniform mycelial growth on the solid substrate surface and enhances the production of secondary metabolites [20,21,22,23].

### 2.3. Extraction and Isolation

After 30 days of fermentation, the fermented solid product was extracted six times with ethyl acetate at room temperature (24 h each), and the combined extracts were concentrated under reduced pressure at 50 °C to constant weight, yielding a crude extract (342.2 g). This crude extract was subsequently fractionated by macroporous resin column chromatography using a methanol-water (0–100%) gradient. The 90% methanol–water eluted fraction (151.0 g) was further separated by silica gel column chromatography with a CH_2_Cl_2_-MeOH gradient (150:1, 100:1, 75:1, 50:1, 25:1, 10:1) to afford seven fractions (A–G). Fraction E (11.3 g) was purified by Sephadex LH-20 (MeOH as mobile phase), yielding five subfractions (E.1–E.5). Subfraction E.2 (6.6 g) was subjected to RP-18 column chromatography and eluted with a MeOH-H_2_O gradient (30:70 to 100:0) to give six subfractions (A–F). Subfraction E.2-F was further separated by repeated silica gel column chromatography using a CH_2_Cl_2_-MeOH gradient (150:1, 100:1, 75:1, 50:1, 25:1, 10:1), yielding seven subfractions (a–g). Subfraction d (34.5 mg) was purified by semi-preparative HPLC with CH_3_CN-H_2_O (45:55, isocratic; 3.0 mL/min; detection at 210 and 254 nm) to afford compound **4** (4.6 mg, t*_R_* = 23 min). Under the same HPLC conditions, subfraction e (53.1 mg) was separated to yield compound **5** (3.4 mg, t*_R_* = 18 min).

Subfraction E.4 (835.5 mg) was separated by reversed-phase C18 column chromatography with a methanol–water gradient (30:70 to 100:0) to afford six fractions (A–F). Fraction E.4-D (39.5 mg) was further purified by semi-preparative high-performance liquid chromatography using a methanol–water solvent system (65:35, isocratic elution) at a flow rate of 3.0 mL/min, with detection at 210 and 254 nm, yielding compound **1** (6.8 mg, t*_R_* = 28 min), compound **2** (5.9 mg, t*_R_* = 26 min), and compound **3** (4.1 mg, t*_R_* = 31 min).

### 2.4. Spectroscopic Data of New Compounds

Penicitrioid A (**1**): yellow powder; [*α*]25 D −6.50 (*c* 0.006, MeOD); UV (MeOD) λ_max_ (log *ε*): 266 (3.45) nm; IR (KBr) *ν_max_*: 2994, 2944, 1766, 1606, 1272, 1046 cm^−1^; HR-TOF-ESI-MS *m*/*z* 178.0863 [M + H]^+^ (calcd for C_10_H_11_NO_2_, 178.0863); ^1^H NMR (500 MHz, MeOD) and ^13^C NMR (126 MHz, MeOD) data; see Table 1.

Penicitrioid B (**2**): white crystals; [*α*]20 D 7.01 (*c* 0.007, MeOD); UV (MeOD) λ_max_ (log *ε*): 269 (3.45) nm; IR (KBr) *ν_max_*: 3016, 2925, 1788, 1744, 1599, 1280 cm^−1^; HR-TOF-ESI-MS *m*/*z* 236.0919 [M + H]^+^ (calcd for C_12_H_13_NO_4_, 236.0919); ^1^H NMR (500 MHz, MeOD) and ^13^C NMR (126 MHz, MeOD) data; see Table 1.

Penicitrioid C (**3**): yellow crystals; [*α*]25 D −4.60 (*c* 0.010, MeOD); UV (MeOD) λ_max_ (log *ε*): 203 (4.08) nm; IR (KBr) ν*_max_*: 3422, 3067, 2936, 2827, 1716, 1609, 1237 cm^−1^; HR-TOF-ESI-MS *m*/*z* 207.1127 [M + H]^+^ (calcd for C_11_H_14_N_2_O_2_, 207.1127); ^1^H NMR (500 MHz, MeOD) and ^13^C NMR (126 MHz, MeOD) data; see Table 1.

### 2.5. ECD Computational Details

The absolute configurations of compounds **1**, **2**, and **3** were established by ECD calculations based on time-dependent density functional theory (TDDFT). Initial conformational searches were carried out in BARISTA (CONFLEX Corporation, Japan) using the MMFF94 force field to explore plausible stereoisomers, and conformers within an energy cutoff of 10 kcal/mol were retained. The selected low-energy conformers were further optimized at the B3LYP/6-31G(d) and B3LYP/6-311+G(d) levels under density functional theory (DFT). Subsequently, ECD computations were performed using Gaussian 09 (Gaussian Inc., USA) [15]. Gas-phase TD-DFT calculations were performed at the B3LYP/6-311+G(d,p) basis set level. Final simulated ECD curves were generated following Boltzmann-weighted averaging over all optimized conformers.

### 2.6. Antifungal Activity Assay

The antifungal activities of compounds **1**–**5**, isolated from *P. citrinum* VDL118 in this work, were evaluated against five plant-pathogenic fungi obtained from the China General Microbiological Culture Collection Center (CGMCC). The tested strains included *G. trabeum*, *C. versicolor*, *F. solani*, *B. cinerea*, and *F. graminearum.*

A broth microdilution method in 96-well plates was employed to determine the MICs of the target compounds. Thiabendazole and carbendazim (purchased from Aladdin Biochemical Technology Co., Ltd., Shanghai, China) were used as positive controls, while an equivalent concentration of dimethyl sulfoxide (DMSO) solution served as the negative control. After incubation at 28 °C for 48 h, the MIC values were recorded.

## 3. Results

### 3.1. Structure Analysis

Compound **1** was isolated as a yellow powder and assigned the molecular formula C_10_H_11_NO_2_ based on positive HR-TOF-ESI-MS (*m*/*z* 178.0863 [M + H]^+^, calcd for 178.0863), combined with the ^13^C NMR spectrum, corresponding to six indices of hydrogen deficiency. The infrared (IR) spectrum exhibited one distinct carbonyl stretching band at 1766 cm^−1^, as well as a band at 2994 cm^−1^ attributable to unsaturated or aromatic C–H stretching vibrations. Analysis of the ^1^H NMR spectrum (Table 1) revealed one aromatic proton at *δ*_H_ 8.74 (1H, s, H-2), one methine proton at *δ*_H_ 5.79 (1H, s, H-5), and three tertiary methyl groups at *δ*_H_ 1.65 (3H, s, H-10), 2.40 (3H, s, H-9), and 2.65 (3H, s, H-8), indicative of a 2,3,4,5-tetrasubstituted pyridine ring. Combined analysis of distortionless enhancement by polarization transfer (DEPT) and heteronuclear single-quantum coherence (HSQC) spectra (Figures S3 and S5) enabled classification of the ^13^C NMR resonances into 10 carbon atoms: one methine proton (*δ*_C_ 79.2), three methyl carbons (*δ*_C_ 19.4, 14.6, and 22.3), five olefinic or conjugated carbons (*δ*_C_ 144.6, 121.5, 159.7, 128.1, and 163.9), and one carbonyl carbon (*δ*_C_ 170.7). The one carbonyl group and three double-bond equivalents accounted for four of the six indices of hydrogen deficiency, with the remaining two indices indicating the presence of two rings in the structure. These data collectively suggested that compound **1** is a furopyridine alkaloid.

**Figure 2:**
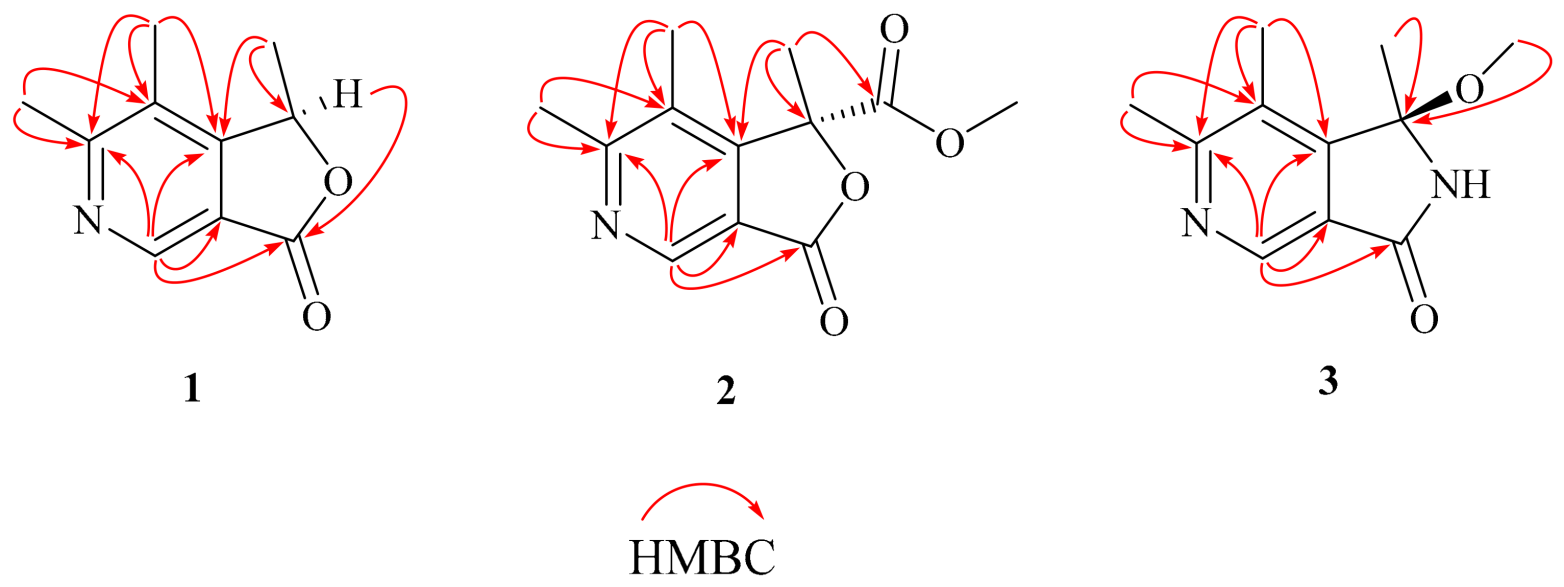
Key HMBC correlations of compounds **1**–**3**.

**Figure 3:**
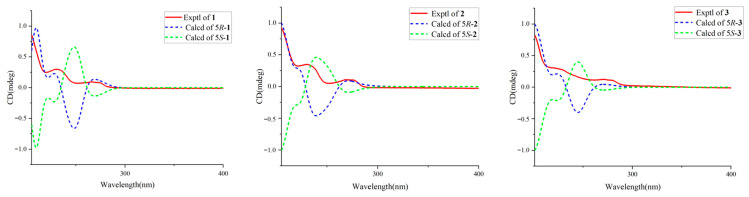
The possible isomers **1**–**3**, the ECD experimental spectra of **1**–**3,** and the calculated ECD spectra of **1**–**3**.

Further analyses of the 2D NMR spectra facilitated elucidation of the planar structure of compound **1**. The core framework of the tetrasubstituted pyridine ring (ring A) was established based on key correlations from H-2 to C-2a, C-3, C-5a, and C-7, from Me-8 to C-7 and C-6, and from Me-9 to C-6, C-5a, and C-7, observed in the heteronuclear multiple-bond correlation (HMBC) spectrum (Figure 2). The fragment of ring B was identified through combined interpretation of chemical shifts and HMBC cross-peaks from Me-10 to C-5 and C-5a, and one methine proton at H-5 with C-3. On the basis of these data, the planar structure of compound **1** was fully determined. The sole stereogenic center, C-5, was assigned the *R* configuration by a combination of experimental TDDFT-calculated ECD spectroscopy. As illustrated in Figure 3, the experimental ECD spectrum closely matched the theoretical spectrum predicted for the 5*R* enantiomer, confirming the *R* configuration at C-5 (Figure 3). Accordingly, the structure of **1** was unambiguously established and named penicitrioid A (Figure 1).

Compound **2**, isolated as a white crystal, was assigned the molecular formula C_12_H_13_NO_4_ based on HR-TOF-ESI-MS ([M + H]^+^ *m*/*z* 236.0919, calcd 236.0919) and ^13^C NMR data, indicating seven degrees of unsaturation. The ^1^H and ^13^C NMR data of compound **2** closely resemble those of **1**, indicating a common core scaffold. However, compound **2** added one oxygenated methyl group and one carbonyl resonance. The fragment of a methyl formate group attached to C-5 was identified through combined interpretation of chemical shifts and HMBC cross-peaks, including from Me-10 to C-11. On the basis of these data, the planar structure of compound **2** was fully determined. The absolute configuration of C-5 was determined as *R* by comparison of the experimental ECD with that calculated (Figure 3). Thus, the structure of **2** was established and designated as penicitrioid B (Figure 1).

Compound **3**, obtained as a yellow crystal, was assigned the molecular formula C_11_H_14_N_2_O_2_ based on HR-TOF-ESI-MS ([M + H]^+^ *m*/*z* 207.1127, calcd 207.1127) and ^13^C NMR data, indicating six degrees of unsaturation. Combined with the characteristic of an N–H stretching vibration at 3422 cm^−1^ observed in its IR spectrum, this information suggested that a pyrrole ring appeared in compound **3** in place of the furan moiety found in **1**, which could be supported by the upfield carbonyl carbon (C-7) from *δ*_C_ 170.7 to 169.3 in **3**. Further comparison of their ^13^C NMR and DEPT spectra, an additional quaternary carbon at *δ*_C_ 92.1 and a methoxy methyl at *δ*_C_ 50.8 appeared in **3** instead of the methine at *δ*_C_ 79.2, suggesting the methoxy group substituted at C-5 in **3**. This suggestion was supported by the HMBC cross-peaks between the MeO and C-5 (Figure 2). Similarly, the absolute configuration of C-5 was assigned as *R* based on the ECD method (Figure 3). Thus, the structure of **3** was established and named penicitrioid C (Figure 1).

### 3.2. The Biosynthetic Pathways of Compounds **1**–**3**

The biosynthesis of penicitrioids A–C (**1**–**3**) is proposed to originate via the polyketide synthase (PKS)-catalyzed condensation of acetyl-coenzyme A (CoA) and malonyl-CoA, forming 4-hydroxy-6-methyl-2H-pyran-2-one [34]. This key intermediate undergoes regioselective methylation at C-6 and C-7 catalyzed by S-adenosylmethionine (SAM)-dependent methyltransferases, followed by prenylation at the appropriate position via prenyltransferase (PT) using dimethylallyl diphosphate (DMAPP). This assembly line constitutes a highly conserved upstream module [34], consistent with the well-established polyketide biosynthetic logic in P. citrinum, such as the polyketide chain elongation mechanism observed in mevastatin biosynthesis. However, significant divergence is observed in the downstream cyclization and post-modification mechanisms among the three compounds; their biosynthetic logic and enzymatic basis are detailed below.

Biosynthetic logic of penicitrioid A (**1**): First, the pyranone ring undergoes aminolysis via nucleophilic substitution of oxygen by nitrogen, yielding 4-hydroxy-3-prenyl-6,7-dimethyl-2H-pyridin-2-one. This oxygen-to-nitrogen exchange is a conserved step in the biosynthesis of fungal pyridine alkaloids and has been documented in the pathways of compounds such as pyridoxatin and tenellin [35,36]. Subsequently, the pyridone nitrogen initiates an intramolecular nucleophilic attack on the prenyl side chain, undergoing nitrogen heterocyclization to construct the 1,1,6,7-tetramethyl-1,2-dihydro-3H-pyrrolo[3,4-c]pyridin-3-one skeleton. Such heterocyclization of prenyl side chains has precedents in *Penicillium* alkaloids; for instance, the biosynthesis of simplicissin from *Penicillium simplicissimum* involves a similar intramolecular cyclization process [37]. Next, a cytochrome P450 monooxygenase (P450) catalyzes the stereoselective oxidation of one methyl group to a hydroxymethyl, establishing the *R* configuration at C-1. Stereoselective oxidations catalyzed by P450 enzymes are highly conserved post-modification steps in fungal secondary metabolism and are widely involved in the structural diversification of *Penicillium* alkaloids [38]. Subsequently, SAM-dependent O-methyltransferase catalyzes methoxylation, followed by dehydrogenase-mediated oxidative dehydrogenation to aromatize the dihydropyrrole ring, ultimately yielding (R)-1,6,7-trimethylfuro[3,4-c]pyridin-3(1H)-one.

Biosynthetic logic of penicitrioid B (**2**): In contrast to penicitrioid A, the biosynthesis of penicitrioid B features oxygen heterocyclization of the prenyl side chain, forming a gem-dimethyl-substituted furo[3,4-c]pyranone core [39]. This oxygen heterocyclization stands in stark contrast to the nitrogen heterocyclization observed in A, and its regioselectivity may be governed by enzymatic conformational control or the orientation of substrate binding. Subsequently, a P450 monooxygenase stereoselectively oxidizes one methyl of the gem-dimethyl group to a carboxyl group—a multi-step oxidation process commonly observed in fungal polyketide biosynthesis, such as the P450 oxidation steps in the biosynthesis of brasilianic acid from *Penicillium brasilianum*. Notably, carboxylation occurs prior to the conversion of the pyranone ring to the pyridone ring, a sequence that differs from most known pyridine alkaloid pathways and suggests the presence of unique enzymatic activities in this strain. Finally, the pyranone is converted to a pyridone through oxygen-to-nitrogen exchange, and the carboxyl group undergoes methyl esterification catalyzed by a SAM-dependent methyltransferase, completing the final product.

**Scheme 1:**
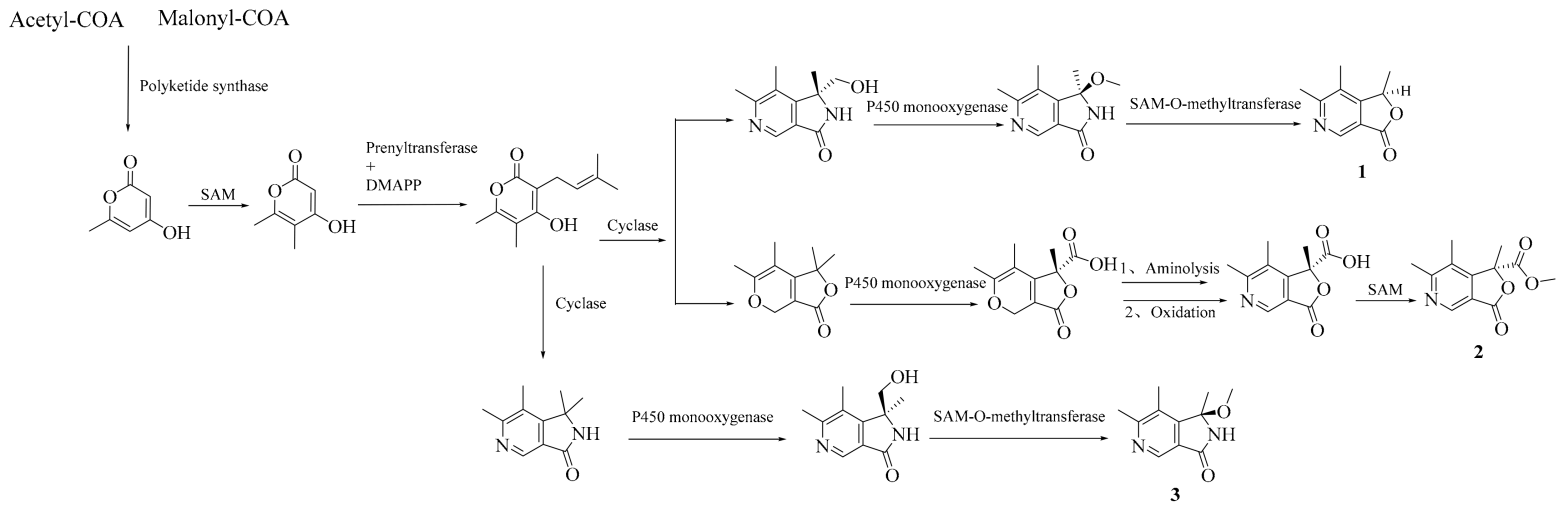
Hypothetical biogenetic pathway of **1**–**3.**

Biosynthetic logic of penicitrioid C (**3**): Penicitrioid C follows a similar but abbreviated pathway compared to penicitrioid A: pyranone-to-pyridone conversion via aminolysis, followed by nitrogen heterocyclization to form the pyrrolo[3,4-c]pyridinone skeleton, and then stereoselective oxidation and methoxylation to yield the R-configured natural product. This streamlined pathway, in comparison with A, suggests that nitrogen heterocyclization or oxygen heterocyclization of its prenyl side chain may represent branching points regulated by competing enzymatic systems. In summary, the biosynthetic pathways of compounds **1**–**3** are based on a conserved PKS upstream module but exhibit remarkable diversity in downstream cyclization and post-modification steps. These transformations are supported by well-established chemical logic and enzymatic precedents and are highly consistent with reported biosynthetic mechanisms of fungal pyridine alkaloids. The discovery of compounds **1**–**3** not only enriches the metabolic product spectrum of P. citrinum but also provides new chemical probes for investigating the unique biosynthetic gene clusters and enzymatic mechanisms in this strain. A schematic diagram of the proposed biosynthetic pathways for compounds **1**–**3** is shown in Scheme 1.

The two identified compounds were determined to be penicipyrroether A (**4**) [40] and GKK1032A2 (**5**) [41] by comparison with the reported NMR and MS data.

### 3.3. Antifungal Activity

The in vitro antifungal activities of compounds **1**–**5** were evaluated against five phytopathogenic fungi—*G. trabeum*, *C. versicolor*, *F. solani*, *B. cinerea*, and *F. graminearum*—using the broth microdilution method in 96-well plates. Thiabendazole and carbendazim were used as positive controls, while an equivalent concentration of DMSO solution served as the negative control. The tested fungal strains were pre-cultured in potato dextrose (PD) broth at 28 °C for 48 h. Subsequently, spore suspensions were prepared and adjusted to a concentration of 1 × 10^6^ colony-forming units (CFU/mL) using potato dextrose broth (PDB). The antifungal activity assay was performed in 96-well plates following the method described in the literature [42], with slight modifications: 120 μL of the compound stock solution was added to the first well; 60 μL of culture medium was added to wells 2–6; 60 μL of 5% DMSO was added to well 7. A volume of 60 μL was transferred from the first well to the second well, mixed thoroughly, and then serially diluted twofold up to the sixth well, from which 60 μL was discarded. Then, 90 μL of the spore suspension was added to wells 1–7, while 150 μL of liquid medium was added to well 8 as the blank control. Positive controls (carbendazim and thiabendazole) were included in each independent experiment. The initial concentration of the compounds was 100 μg/mL, and after five serial twofold dilutions, the final concentrations ranged from 3.1 to 100 μg/mL. All compounds were tested in triplicate. The plates were incubated statically at 28 ± 0.5 °C for 48 h, after which fungal growth was observed. The MIC was defined as the lowest concentration of the compound that completely inhibited visible fungal growth.

The antifungal activities of compounds **1**–**5** against five phytopathogenic fungi are summarized in Table 2. The results show that all tested compounds exhibited varying degrees of inhibitory activity against the five target strains, with MIC values ranging from 3.1 to 100 μg/mL (Table 2), demonstrating broad-spectrum and significant antifungal properties. To evaluate their activity levels more objectively, this study established the following grading criteria with reference to the general standards for evaluating the antifungal activity of natural products [43,44]: strong activity for MIC ≤ 10 μg/mL, moderate activity for MIC values of 10–100 μg/mL, and weak or no activity for MIC > 100 μg/mL. Accordingly, the antifungal activities of compounds **1**–**5** were classified as follows: compounds **1** and **2** showed strong activity against *G. trabeum*, *C. versicolor*, and *B. cinerea*, with MIC values ranging from 3.1 to 12.5 μg/mL; compounds **1**, **3** and **4** displayed moderate activity against *F. solani* and *F. graminearum* (MIC = 25–50 μg/mL), whereas compound **5** showed weak activity against *F. solani* and *F. graminearum* (MIC > 100 μg/mL). Further analysis revealed that compounds **1**–**4** exerted excellent inhibitory effects against *G. trabeum* (MIC = 3.1 μg/mL), with activity comparable to that of the positive controls carbendazim and thiabendazole. The inhibitory activity against *C. versicolor* was particularly prominent, and compounds **1** and **5** (MIC = 3.1 μg/mL) showed better fungistatic effects than the two positive control drugs. In addition, compound **2** exhibited strong inhibitory activity against *F. solani* (MIC = 3.1 μg/mL), which was superior to carbendazim and equivalent to thiabendazole. Similarly, compounds **1** and **3** also showed strong inhibitory activity against *B. cinerea* (MIC = 3.1 μg/mL), with effects comparable to both positive controls. Based on the above results, compounds **1**–**5** possess certain broad-spectrum antifungal activity against phytopathogenic fungi. Some compounds exhibit relatively significant inhibitory effects on specific strains, indicating their preliminary potential as lead candidates for novel agricultural antifungal agents. 

## 4. Discussion

To date, most alkaloids in nature have been known to be of plant origin, whereas reports on fungal-derived alkaloids—especially those with novel pyridine skeletons—remain relatively scarce [24,25]. In this study, three new pyridine alkaloids were isolated for the first time from *P. citrinum* VDL118, and their structures differ significantly from most previously reported fungal-derived pyridine alkaloids [34,35]. For instance, compared with the furo[3,4-b]pyridine derivatives isolated from *Penicillium* fungus ZZ1751 [36], the latter lack the dihydrofuran moiety and 3-oxo substituent characteristic of compounds **1** and **2**, structural features that have not been previously reported in fungal metabolites. Similarly, the skeleton of compound **3** differs markedly from the pyrrolo[3,4-b]pyridine analogues isolated from *Xylaria* fungi in the pyrrole ring fusion site [37], further highlighting the structural novelty of compound **3**. Combined with the novel sesquiterpenes [14] and alkaloids [15] previously discovered by our group from other endophytic fungi of the same host (*V. dunalianum*), the results of this study further confirm that endophytic fungi from *V. dunalianum* represent a valuable resource reservoir for discovering novel bioactive natural products [11]. Among the isolated compounds **1**–**5**, compounds **1**, **2**, and **5** exhibited stronger inhibitory activity against some plant pathogenic fungi than that of the positive control agents. Compared with structurally similar antifungal pyridine alkaloids (e.g., the furo[2,3-c]pyridine derivatives isolated from *Aspergillus fumigatus* [38], with MIC values of 6.2–12.5 μg/mL against common plant pathogenic fungi), compounds **1**–**3** displayed significantly enhanced antifungal activity, further verifying their structural and functional novelty. These results are consistent with our previous findings [33] that polyyne glycosides isolated from *Xylaria* sp. VDL4 showed better inhibitory effects against *Fusarium oxysporum* and *Phytophthora capsici* than the positive control drugs. This indicates that endophytic fungi from *V. dunalianum* can produce highly potent and broad-spectrum antifungal lead compounds with promising potential for agricultural development.

## 5. Conclusions

In summary, three novel pyridine alkaloids (penicillicitrins A–C, **1**–**3**) and two known compounds from the endophytic fungus *P. citrinum* VDL118 derived from *V. dunalianum* Wight were isolated in this study. Compounds **1**–**3** possess rare dihydrofuro[3,4-c]pyridine and pyrrolo[3,4-c]pyridine skeletons, which not only enrich the structural diversity of fungal alkaloids but also provide a new reference for research on the biosynthesis of polyketide-derived alkaloids in the genus *Penicillium*. All isolated compounds exhibited potent and broad-spectrum antifungal activity against various plant pathogenic fungi. Among them, compounds **1** and **5** showed stronger inhibitory activity against *C. versicolor* (MIC = 3.1 μg/mL) than thiabendazole (MIC = 6.8 μg/mL). The inhibitory effect of compound **2** against *F. solani* (MIC = 3.1 μg/mL) was equivalent to that of carbendazim, indicating promising potential as a lead compound for environmentally friendly agricultural fungicides. This study further demonstrates that endophytic fungi from medicinal plants are valuable resources for discovering natural products with novel structures and significant biological activities, and it provides a solid theoretical and experimental basis for the development of green agricultural chemicals based on endophytic fungal metabolites.

## Figures and Tables

**Table 1:** ^1^H (500 MHz) and ^13^C-NMR (126 MHz) data of compounds **1**–**3** in MeOD (*δ* in ppm). ^a^ *δ* in ppm, *J* in Hz (given in parentheses).

Position	1 ^a^ *δ*_H_ (*J* in Hz)	*δ*_C_	2 ^a^ *δ*_H_ (*J* in Hz)	*δ*_C_	3 ^a^ *δ*_H_ (*J* in Hz)	*δ*_C_
1	-	-	-	-	-	-
2	8.74	144.6 CH	8.80	145.0 CH	8.62	142.1 CH
2a	-	121.5C	-	121.1 C	-	127.5 C
3	-	170.7 C	-	170.0 C	-	169.3 C
4	-	-	-	-	-	-
5	5.79, qd (6.7)	79.2 CH	-	86.7 C	-	92.1 C
5a	-	159.7 C	-	156.6 C	-	153.4 C
5-OCH_3_	-	-	-	-	2.97 s	50.8 CH_3_
6	-	128.1 C	-	128.7 C	-	129.7 C
7	-	163.9 C	-	165.4 C	-	163.6 C
8	2.65 s	22.3 CH_3_	2.67 s	21.3 CH_3_	2.63 s	22.3 CH_3_
9	2.40 s	14.6 CH_3_	2.40 s	14.4 CH_3_	2.46 s	13.6 CH_3_
10	1.65 d (6.7)	19.4 CH_3_	1.94 s	22.7 CH_3_	1.78 s	25.1 CH_3_
11	-	-	-	169.3 C	-	-
11-OCH_3_	-	-	3.71 s	54.2 CH_3_	-	-

**Table 2:** Antifungal activity of compounds **1**–**5** (MIC, μg/mL).

Compd.	MIC (μg/mL)				
	*G. trabeum*	*C. versicolor*	*F. solani*	*B. cinerea*	*F. graminearum*
**1**	3.1	3.1	25.0	3.1	50.0
**2**	3.1	12.5	3.1	12.5	12.5
**3**	3.1	12.5	25.0	3.1	50.0
**4**	3.1	12.5	25.0	50.0	25.0
**5**	6.8	3.1	100.0	25.0	100.0
^a^ Thiabendazole	3.1	6.8	6.8	3.1	6.8
^a^ Carbendazim	3.1	12.5	3.1	3.1	3.1

^a^ Thiabendazole and carbendazim were used as a fungal positive control.

## Data Availability

Data are contained within the article and Supplementary Materials.

## Supplementary Materials

The following supporting information can be downloaded at: https://www.mdpi.com/article/10.3390/jof12040296/s1, spectroscopic data of compounds **1**–**3** (^1^H NMR, ^13^C-DEPT, HSQC, HMBC, ^1^H−^1^H COSY, NOESY, HR-TOF-ESIMS, UV, IR, optical rotation and CD); and supplementary results of the antibacterial activity assays (in image format).
