# Supplementary material for: Novel Antifungal Pyridine Alkaloids from Endophytic Fungus Penicillium citrinum VDL118
Source: J Fungi (Basel). 2026 Apr 20;12(4):296. doi: 10.3390/jof12040296 (PMC13117981; doi:10.3390/jof12040296)

Novel Antifungal Pyridine Alkaloids from Endophytic Fungus *Penicillium citrinum* VDL118

Mei Yang [1,†], Shan Hu [1,†], Zhi-Yu Zhang [1], Fa-Zhong Yang [1], Xiao-Qin Yang [1], Si-Da Xie [1], Ying-Jun Zhang [2], Ping Zhao [1,*] and Guo-Lei Zhu [1,*]

[1] Key Laboratory of State Forestry and Grassland Administration on Highly-Efficient Utilization of Forestry Biomass Resources in Southwest China, Southwest Forestry University, Kunming 650233, China; 17787982850@163.com (M.Y.); hushan@163.com (S.H.); zzy-529_654@swfu.edu.cn (Z.-Y.Z.); yangfazhong105@163.com (F.-Z.Y.); yangxiaoqin@swfu.edu.cn (X.-Q.Y.); dream102035@163.com (S.-D.X.)

[2] Key Laboratory of Phytochemistry and Natural Medicines, Kunming Institute of Botany, Chinese Academy of Sciences, Kunming 650201, China; zhangyj@mail.kib.ac.cn

* Correspondence: hypzhao2022@163.com (P.Z.); guoleizhu@163.com (G.-L.Z.)

† These authors contributed equally to this work.

ORCID[iD]

Ping Zhao: 0000-0002-9652-1235

Guo-Lei Zhu: 0000-0003-0318-7408

Notes

The authors declare no competing financial interest.

Supporting Information

CONTENTS

Figure S1. ^{1}H NMR spectrum of compound **1** in MeOD. 1

Figure S2. ^{13}C NMR spectrum of compound **1** in MeOD. 1

Figure S3. DEPT spectrum of compound **1** in MeOD. 2

Figure S4. ^{1}H-^{1}H COSY spectrum of compound **1** in MeOD. 2

Figure S5. HSQC spectrum of compound **1** in MeOD. 3

Figure S6. HMBC spectrum of compound **1** in MeOD. 3

Figure S7. NOESY spectrum of compound **1** in MeOD. 4

Figure S8. HR-TOF-ESI-MS spectrum of compound **1**. 5

Figure S9. UV spectrum of compound **1**. 6

Figure S10. IR spectrum of compound **1**. 7

Figure S11. Optical rotation spectrum of compound **1**. 7

Figure S 12. CD spectrum of compound **1**. 8

Figure S13. ^{1}H NMR spectrum of compound **2** in MeOD. 9

Figure S14. ^{13}C NMR spectrum of compound **2** in MeOD. 9

Figure S15. DEPT spectrum of compound **2** in MeOD. 10

Figure S16. ^{1}H-^{1}H COSY spectrum of compound **2** in MeOD. 10

Figure S17. HSQC spectrum of compound **2** in MeOD. 11

Figure S18. HMBC spectrum of compound **2** in MeOD. 11

Figure S19. NOESY spectrum of compound **2** in MeOD. 12

Figure S20. HR-TOF-ESI-MS spectrum of compound **2**. 13

Figure S21. UV spectrum of compound **2**. 14
Figure S22. IR spectrum of compound **2**. 15
Figure S23. Optical rotation spectrum of compound **2**. 15
Figure S24. CD spectrum of compound **2**. 16
Figure S25. ^{1}H NMR spectrum of compound **3** in MeOD. 17
Figure S26. 13C NMR spectrum of compound **3** in MeOD. 17
Figure S27. DEPT spectrum of compound **3** in MeOD. 18
Figure S28. ^{1}H-^{1}H COSY spectrum of compound **3** in MeOD. 18
Figure S29. HSQC spectrum of compound **3** in MeOD. 19
Figure S30. HMBC spectrum of compound **3** in MeOD. 19
Figure S31. NOESY spectrum of compound **3** in MeOD. 20
Figure S32. HR-TOF-ESI-MS spectrum of compound **3** in MeOD. 21
Figure S33. UV spectrum of compound **3**. 22
Figure S34. IR spectrum of compound **3**. 23
Figure S35. Optical rotation spectrum of compound **3**. 23
Figure S36. CD spectrum of compound **3**. 24
Figure S37. Fungal inhibition experiment of compounds **1-5**. 24

Figure S1. ^{1}H NMR spectrum of compound **1** in MeOD.

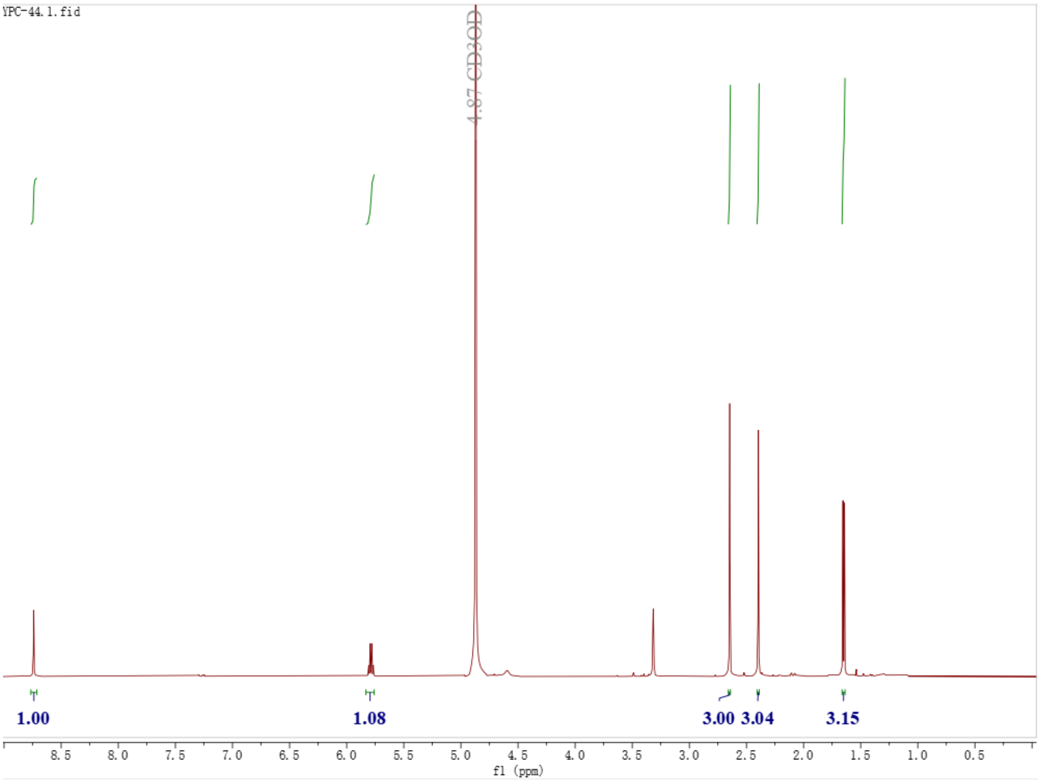

Figure S2. ^{13}C NMR spectrum of compound **1** in MeOD.

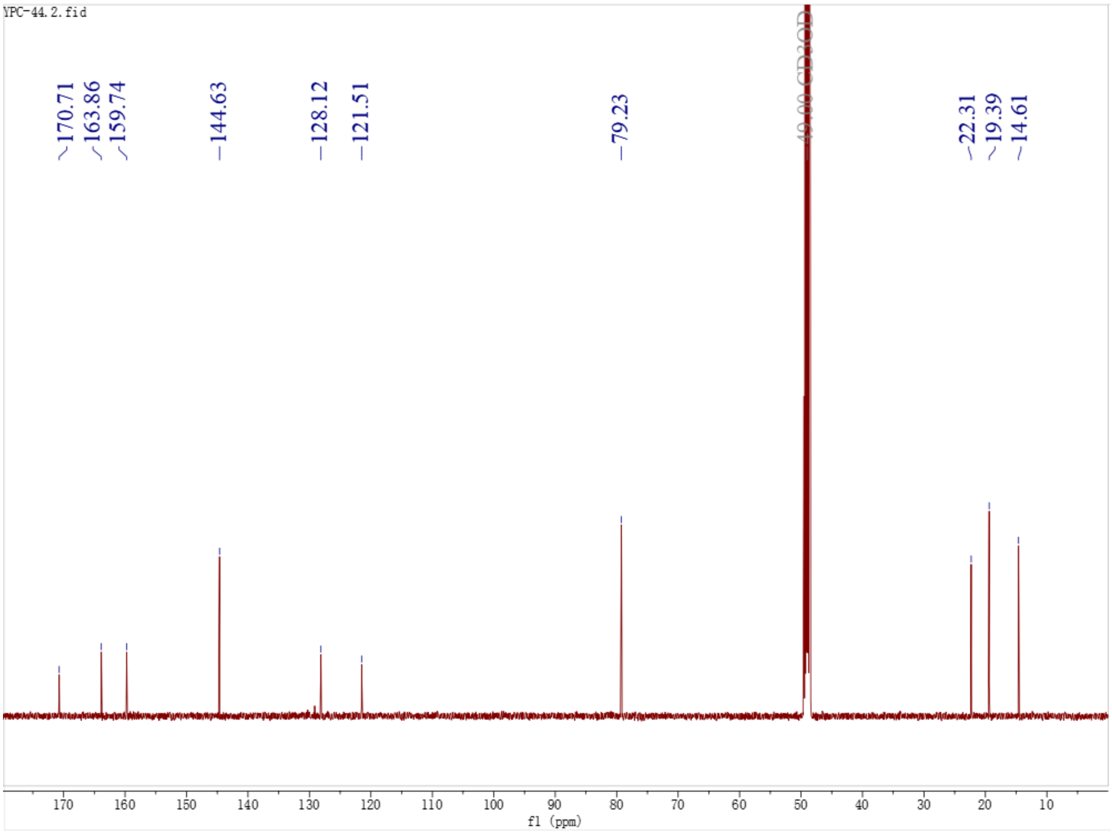

Figure S3. DEPT spectrum of compound **1** in MeOD.

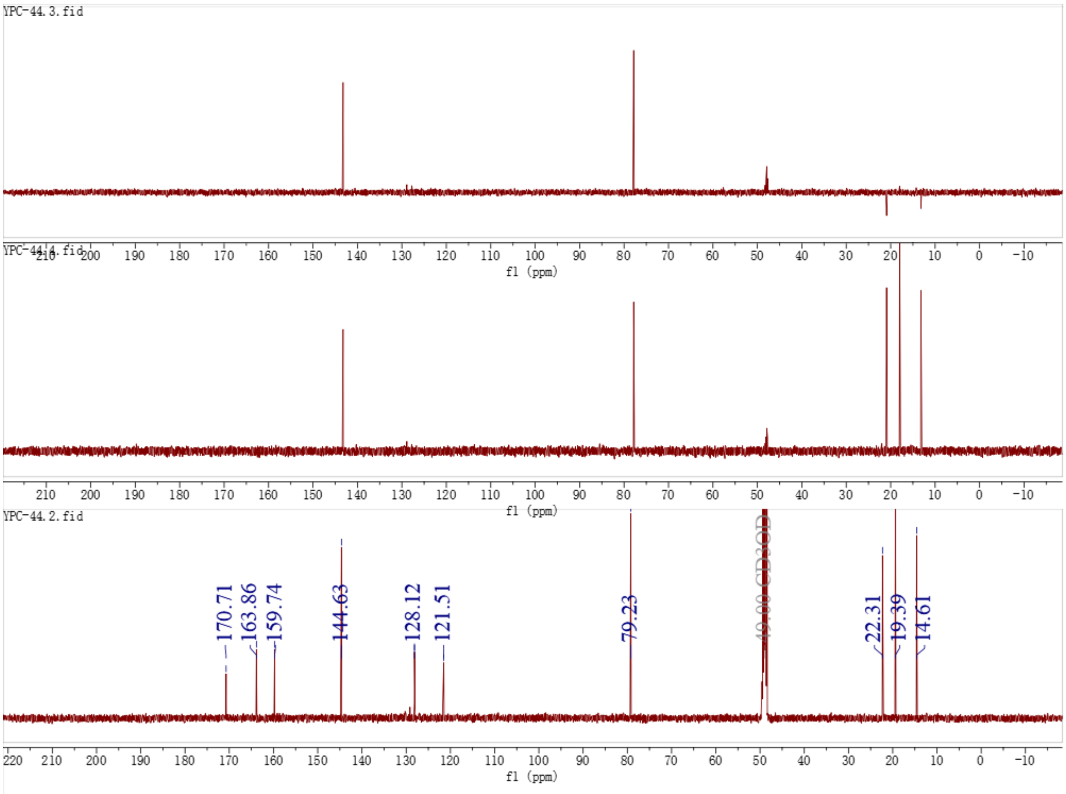

Figure S4. ^{1}H-^{1}H COSY spectrum of compound **1** in MeOD.

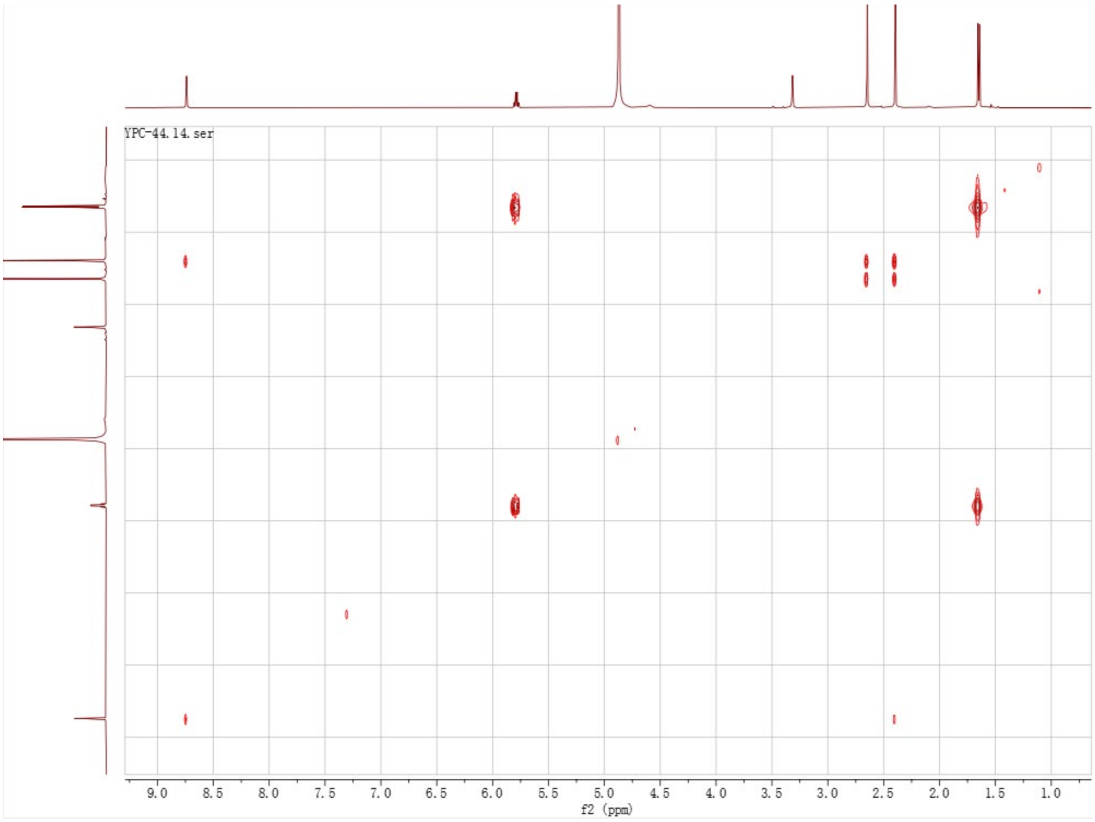

Figure S5. HSQC spectrum of compound **1** in MeOD.

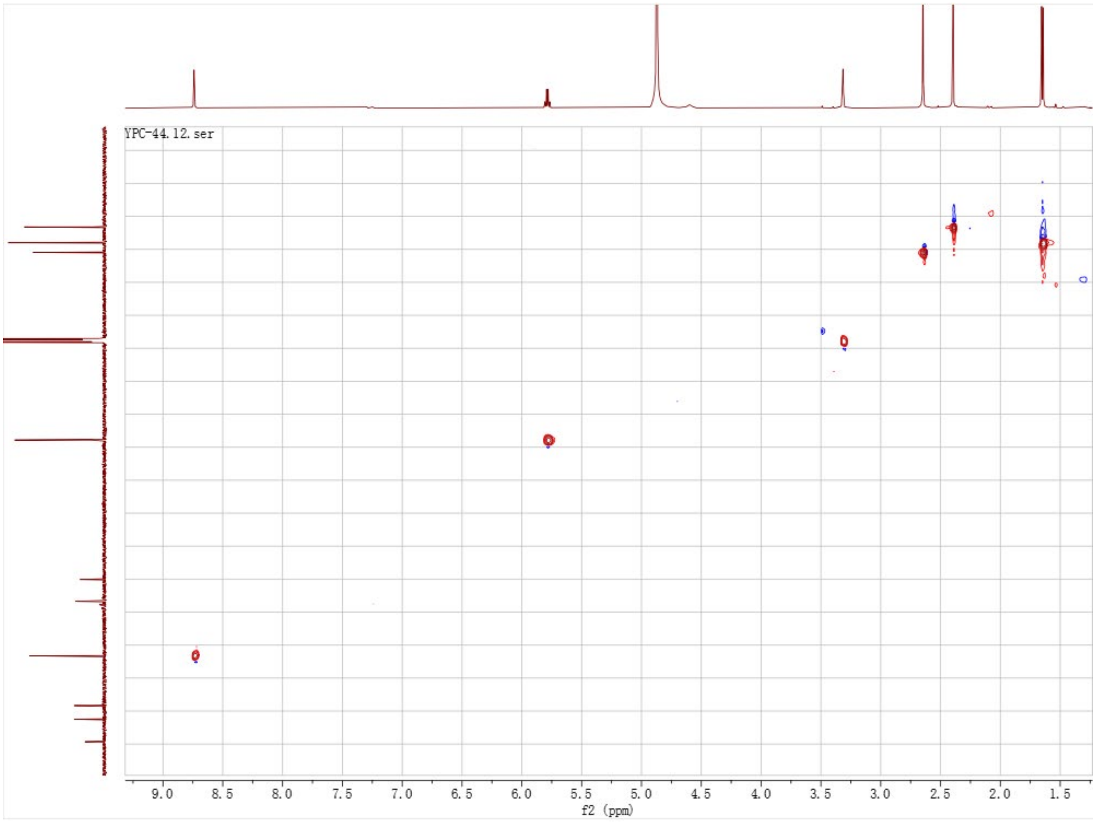

Figure S6. HMBC spectrum of compound **1** in MeOD.

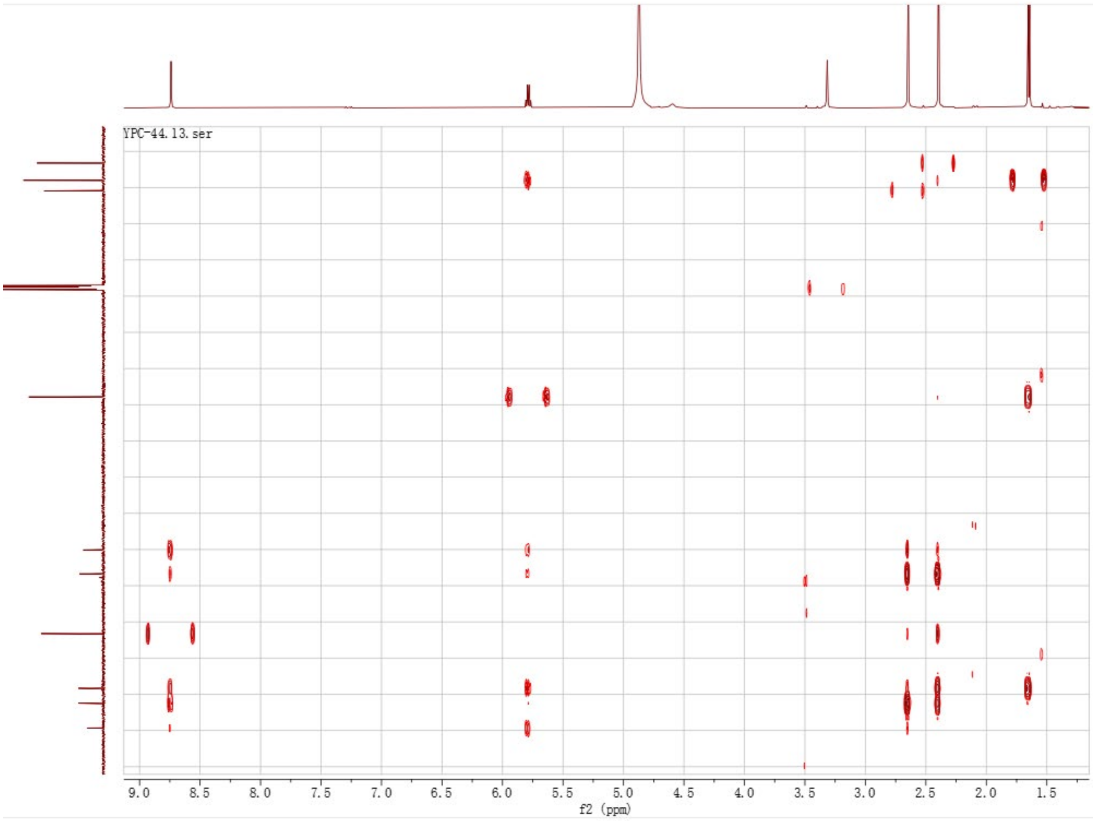

Figure S7. NOESY spectrum of compound **1** in MeOD.

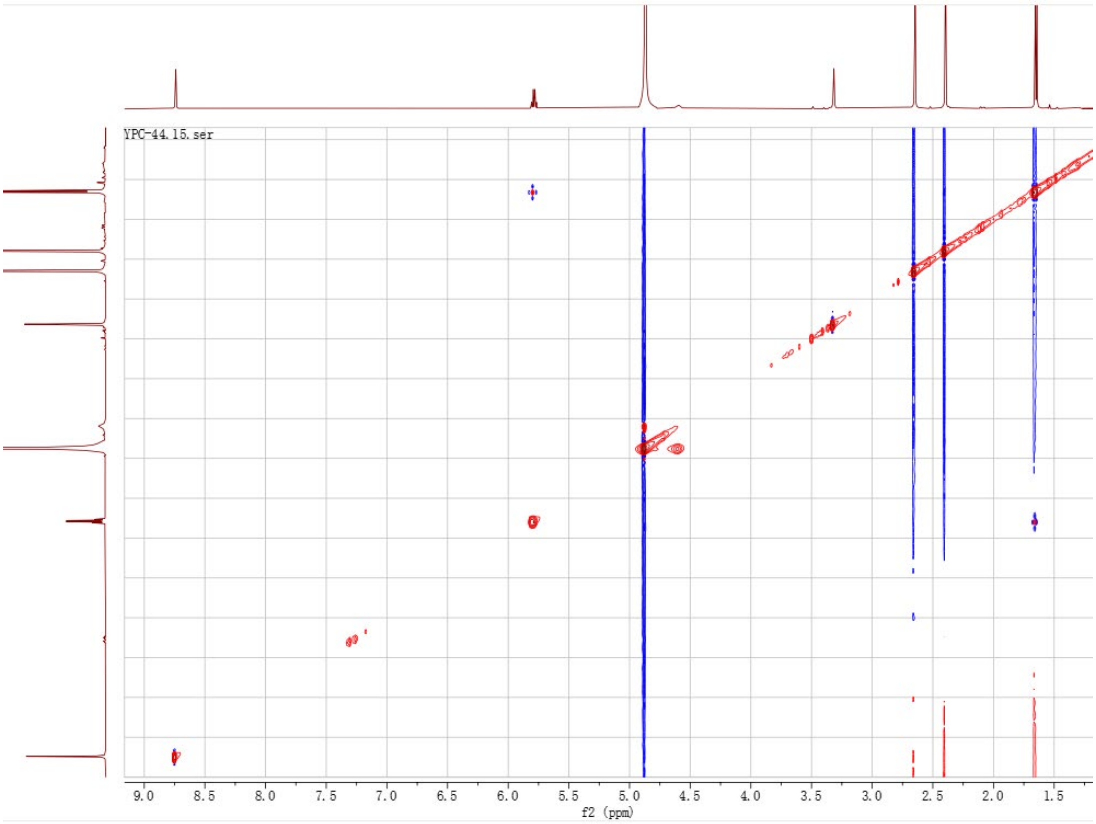

Figure S8. HR-TOF-ESI-MS spectrum of compound **1**.

Qualitative Analysis Report

**Data Filename**	Ypc-44.d	**Sample Name**	Ypc-44
**Sample Type**	Sample	**Position**	Vial 22
**Instrument Name**	Agilent G6230 TOF MS	**User Name**	
**Acq Method**	ESI.m	**Acquired Time**	6/11/2025 4:33:20 PM
**IRM Calibration Status**	Success	**DA Method**	ESI.m
**Comment**			

**Sample Group**		**Info.**
**Acquisition SW Version**	6200 series TOF/6500 series Q-TOF B.05.01 (B5125.2)	

User Spectra

Fragmentor Voltage	Collision Energy	Ionization Mode
200	0	ESI

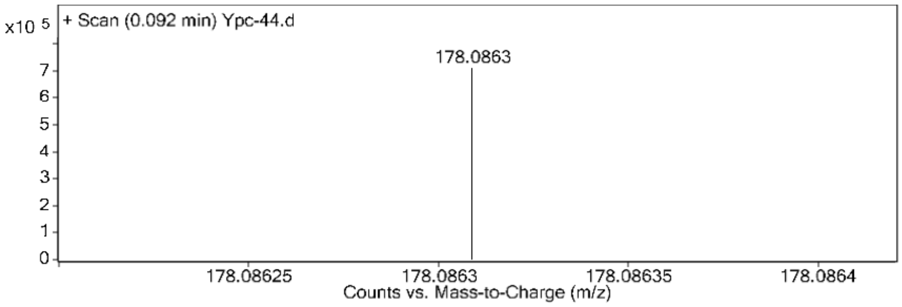

Peak List

m/z	z	Abund	Formula	Ion
107.0733	1	182147.05		
121.0509	1	270794.25		
135.0677	1	43613.16		
163.0623	1	54639.09		
178.0863	1	708484	C10 H12 N O2	M+
179.0893	1	76427.65	C10 H12 N O2	M+
393.2976	1	310261.06		
394.3009	1	70662.04		
922.0098	1	340389.22		
923.0123	1	63131.71		

Formula Calculator Element Limits

Element	Min	Max
C	0	200
H	0	400
O	0	10
N	1	1

Formula Calculator Results

Formula	CalculatedMass	Mz	Diff.(mDa)	Diff. (ppm)	DBE
C10 H12 N O2	178.0868	178.0863	0.5	2.8	5.5

Figure S9. UV spectrum of compound **1**.

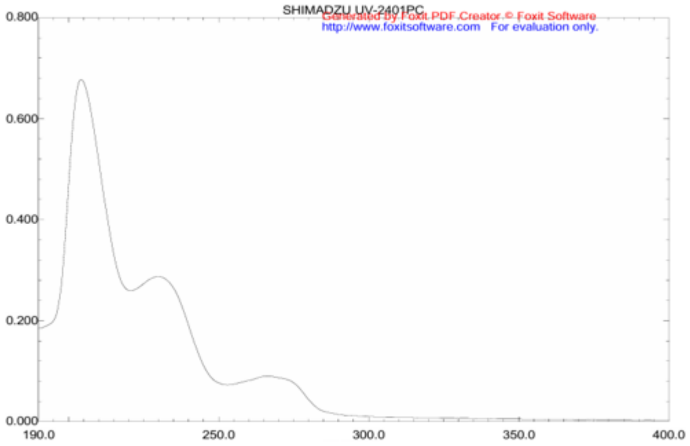

Generated by Foxit PDF Creator © Foxit Software
http://www.foxitsoftware.com For evaluation only.

File Name: YPC-44
Created At: 18:07 25-10-17

YPC-44
Sample Concentration: 0.0057 mg/mL
Solvent: Methanol

Data: Raw
Measurement Mode: Abs. (Absorbance)
Scan Speed: Medium
Slit Width: 5.0
Sampling Interval: 0.5

No.	Wavelength (nm.)	Abs.
1	266.50	0.0898
2	230.00	0.2868
3	204.00	0.6770

Figure S10. IR spectrum of compound **1**.

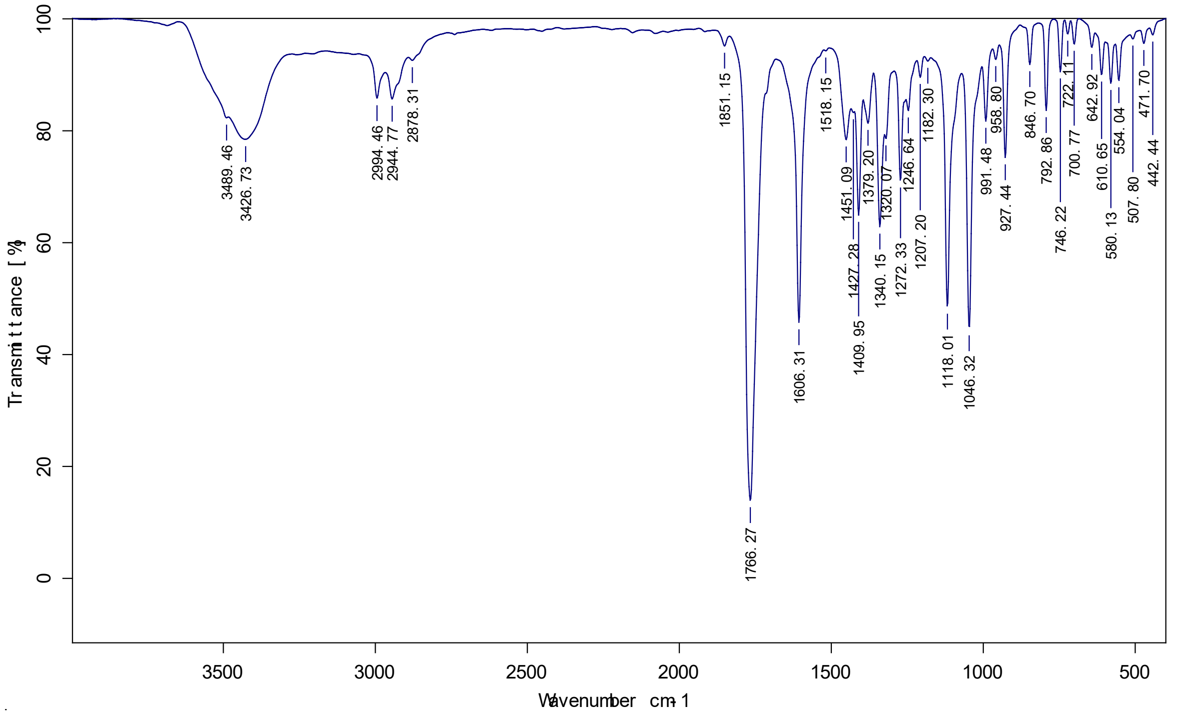

Sample Name: Ypc-44	Resolution: 4	Beamsplitter Setting: KBr
Sample Form: KBr	Aperture Setting: 6 mm	Source Setting: MIR
Path of File: E:\data	Number of Background Scans: 16	Instrument Type: BRUKER VERTEX 70
Date of Measurement: 2025/10/16	Number of Sample Scans: 16	Soft Version: OPUS8.1

Figure S11. Optical rotation spectrum of compound **1**.

Rudolph Research Analytical

This sample was measured on an Autopol VI, Serial #91058
Manufactured by Rudolph Research Analytical, Hackettstown, NJ, USA.

Measurement Date : Sunday, 19-OCT-2025

Set Temperature : 25.0

Time Delay : Disabled

Delay between Measurement : Disabled

n	Average	Std.Dev.	% RSD	Maximum	Minimum
5	-6.50	0.66	-10.15	-6.02	-7.23

S.No	Sample ID	Time	Result	Scale	OR °Arc	WLG.nm	Lg.mm	Conc.g/100ml	Temp.
1	YPC-44	03:16:24 PM	-6.02	SR	-0.005	589	100.00	0.083	25.0
2	YPC-44	03:16:30 PM	-7.23	SR	-0.006	589	100.00	0.083	25.0
3	YPC-44	03:16:37 PM	-6.02	SR	-0.005	589	100.00	0.083	25.0
4	YPC-44	03:16:43 PM	-6.02	SR	-0.005	589	100.00	0.083	25.0
5	YPC-44	03:16:50 PM	-7.23	SR	-0.006	589	100.00	0.083	25.0

Figure S 12. CD spectrum of compound **1**.

YPC-44

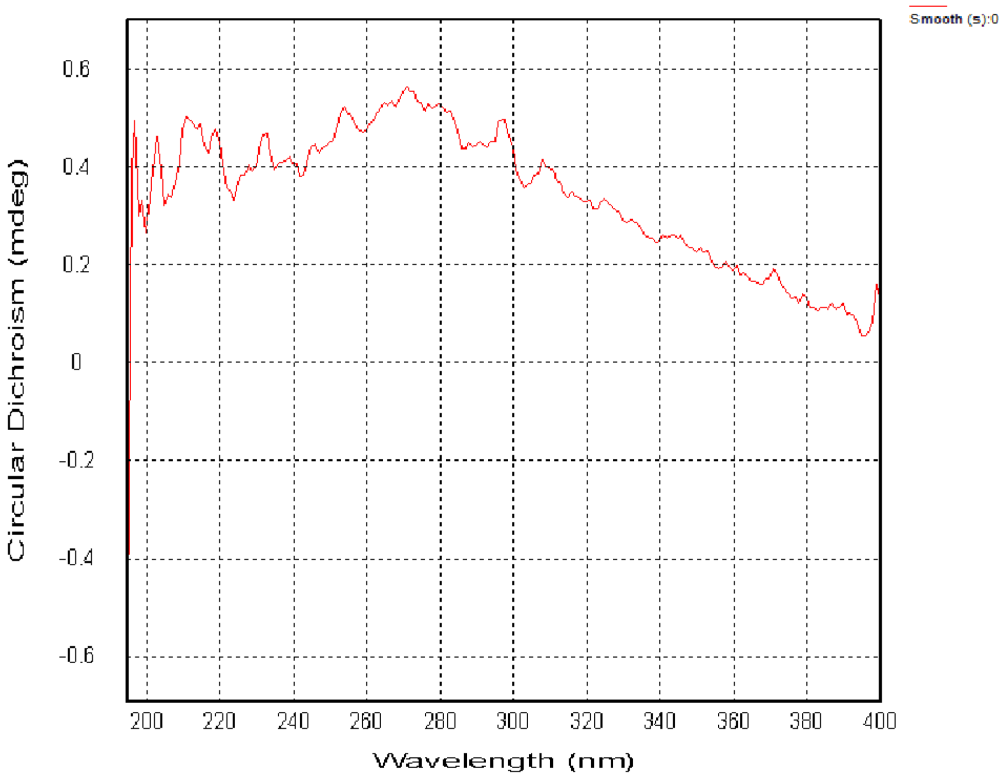

Figure S13. ^{1}H NMR spectrum of compound **2** in MeOD.

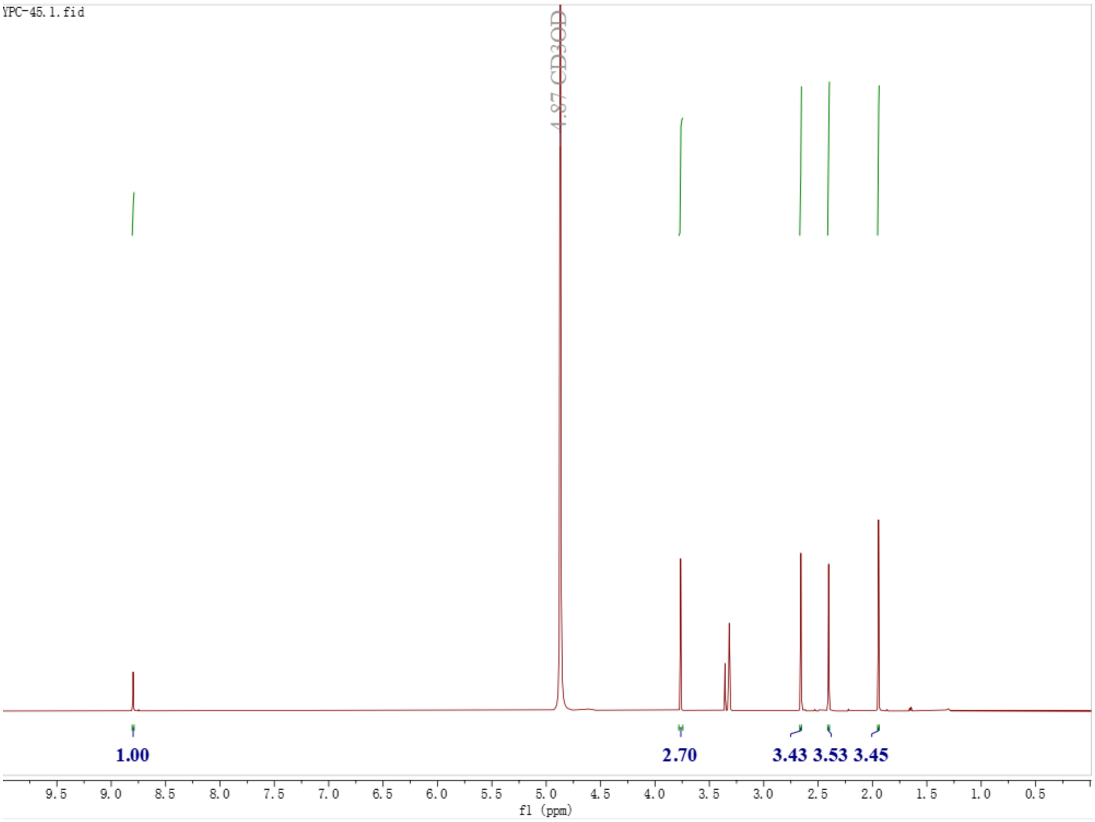

Figure S14. ^{13}C NMR spectrum of compound **2** in MeOD.

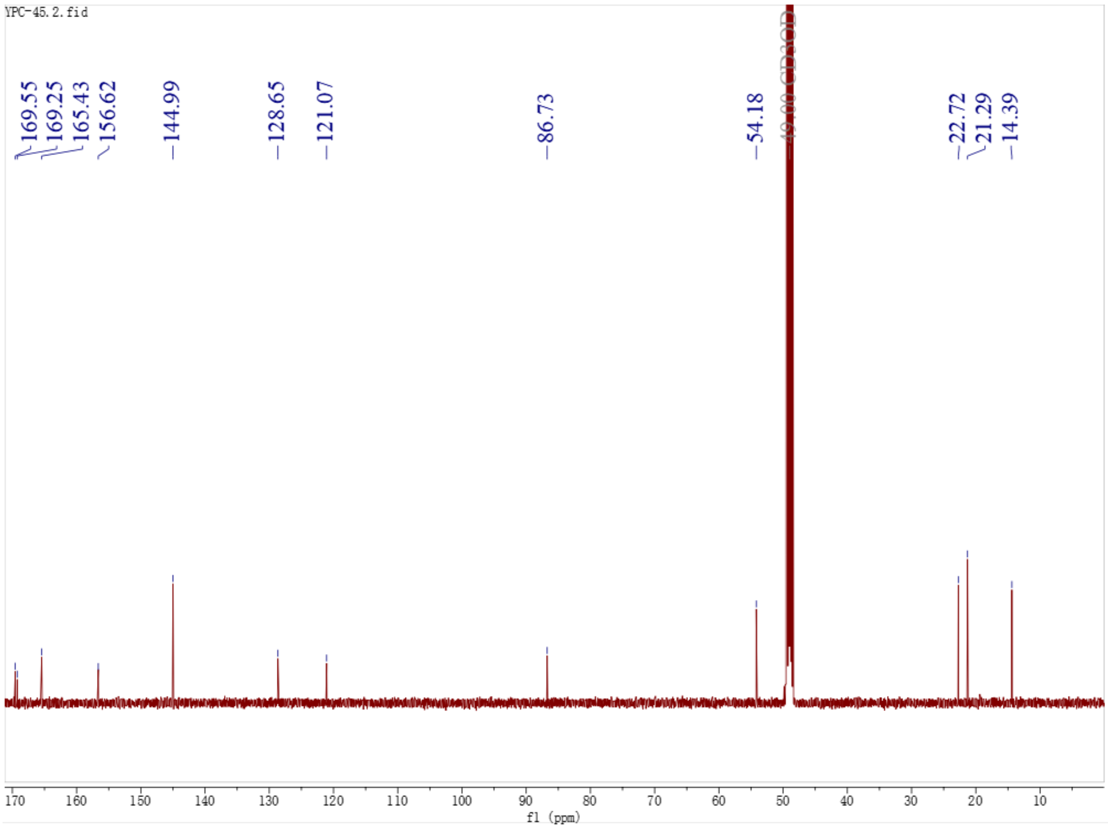

Figure S15. DEPT spectrum of compound **2** in MeOD.

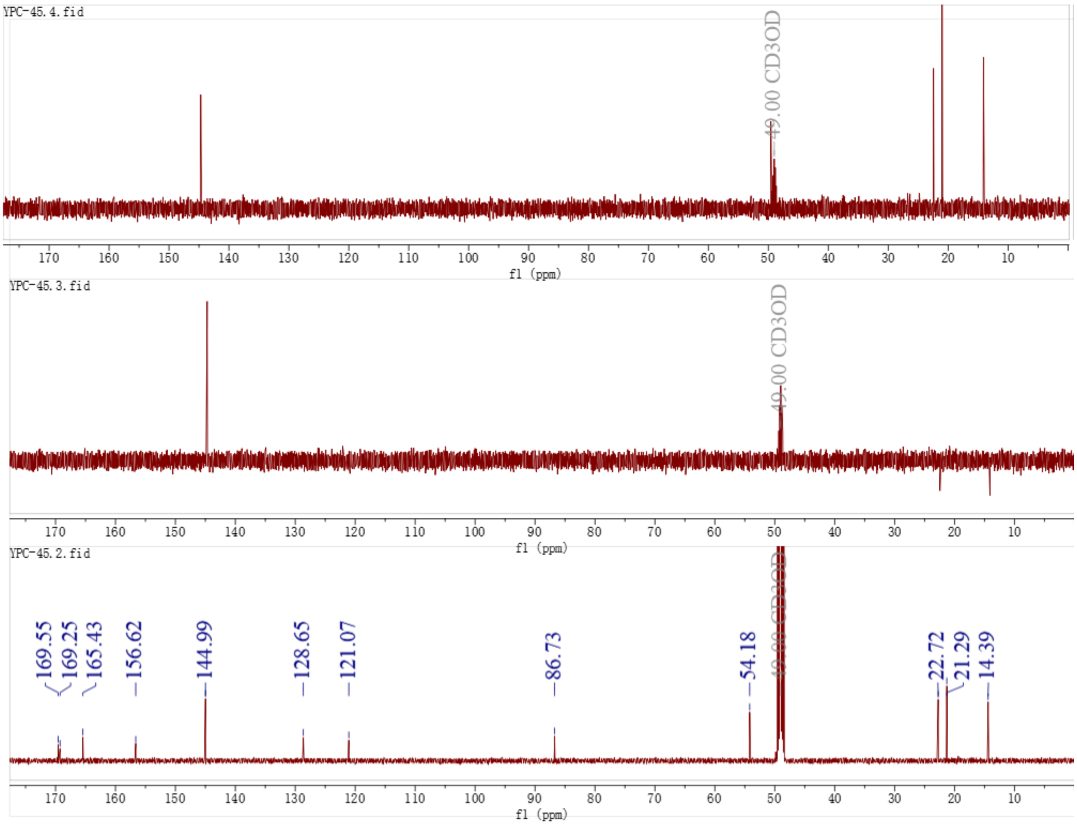

Figure S16. ^{1}H-^{1}H COSY spectrum of compound **2** in MeOD.

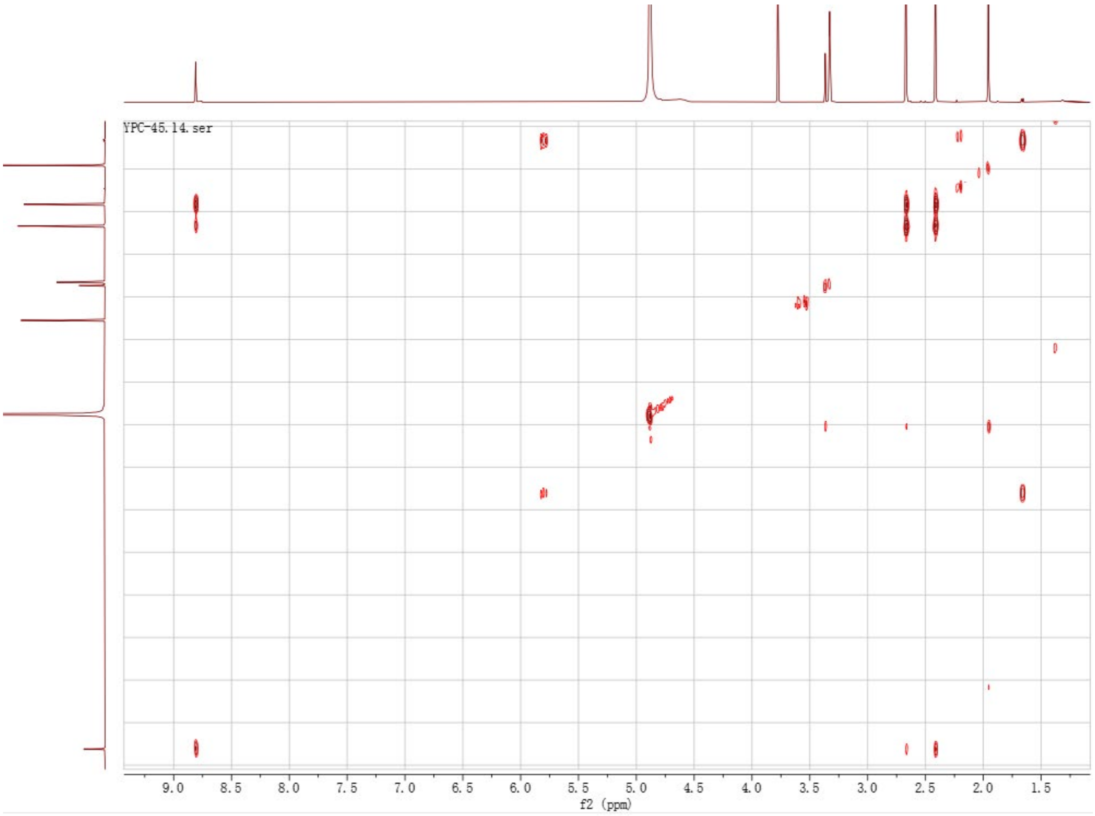

Figure S17. HSQC spectrum of compound **2** in MeOD.

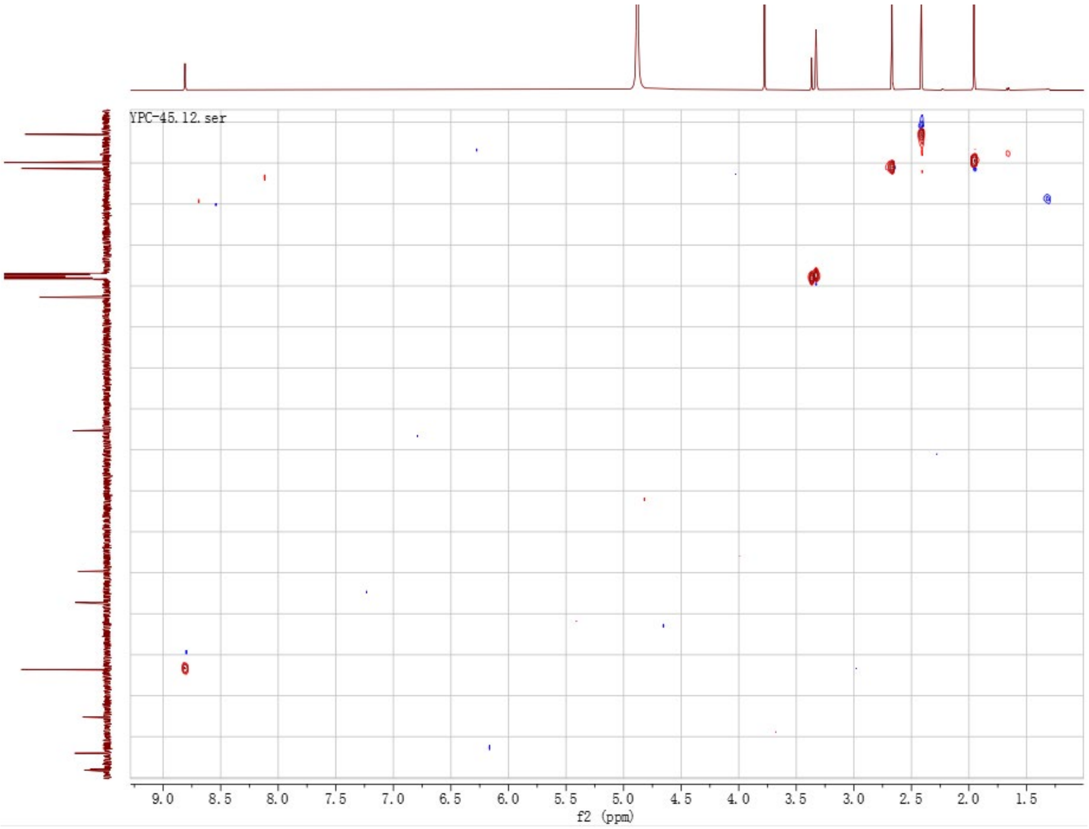

Figure S18. HMBC spectrum of compound **2** in MeOD.

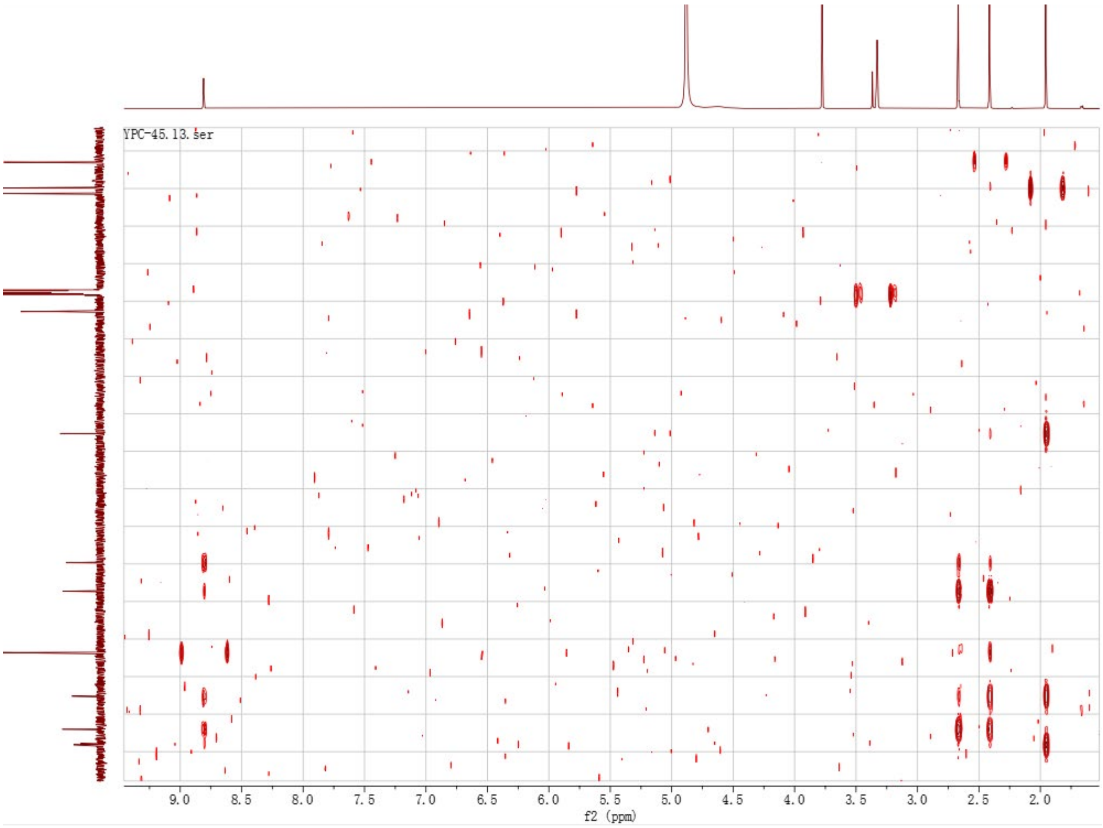

Figure S19. NOESY spectrum of compound **2** in MeOD.

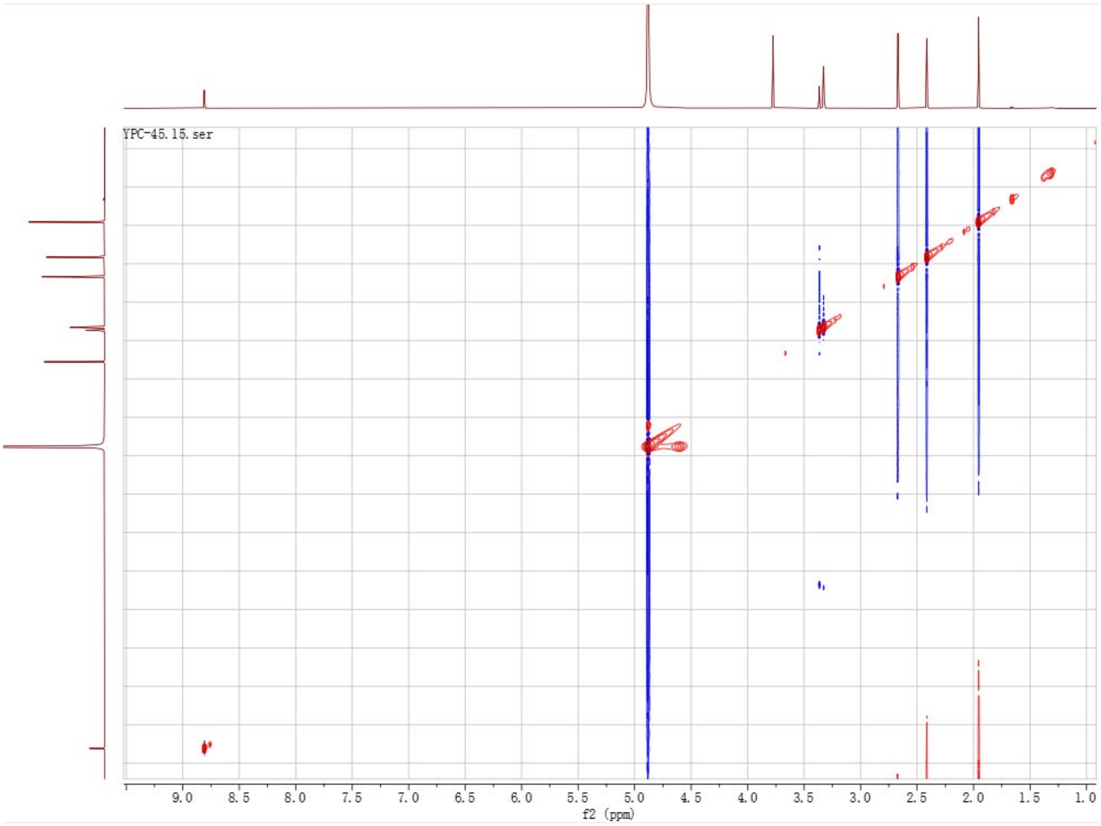

Figure S20. HR-TOF-ESI-MS spectrum of compound **2**.

Qualitative Analysis Report

**Data Filename**	Ypc-45.d	**Sample Name**	Ypc-45
**Sample Type**	Sample	**Position**	Vial 23
**Instrument Name**	Agilent G6230 TOF MS	**User Name**	
**Acq Method**	ESI.m	**Acquired Time**	6/11/2025 4:34:41 PM
**IRM Calibration Status**	Success	**DA Method**	ESI.m
**Comment**			

Sample Group **Info.**
Acquisition SW 6200 series TOF/6500 series
Version Q-TOF B.05.01 (B5125.2)

User Spectra

Fragmentor Voltage	Collision Energy	Ionization Mode
200	0	ESI

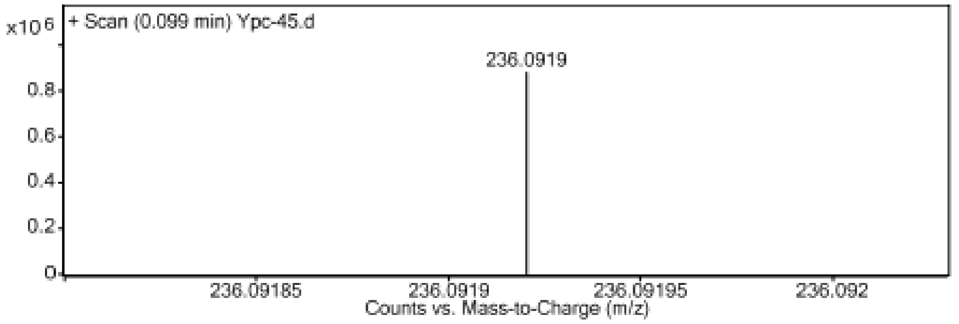

Peak List

m/z	z	Abund	Formula	Ion
107.0731	1	106863.86		
121.0509	1	268375.72		
121.0866	1	139339.48		
149.083	1	100300.74		
177.0782		244904.69		
208.0965	1	200401.33		
236.0919	1	880859.38	C12 H14 N O4	M+
237.0949	1	115603.42	C12 H14 N O4	M+
393.2975	1	112783.55		
922.0098	1	326693.31		

Formula Calculator Element Limits

Element	Min	Max
C	0	200
H	0	400
O	1	10
N	1	1

Formula Calculator Results

Formula	CalculatedMass	Mz	Diff.(mDa)	Diff. (ppm)	DBE
C12 H14 N O4	236.0923	236.0919	0.4	1.6	6.5

--- End Of Report ---

Figure S21. UV spectrum of compound **2**.

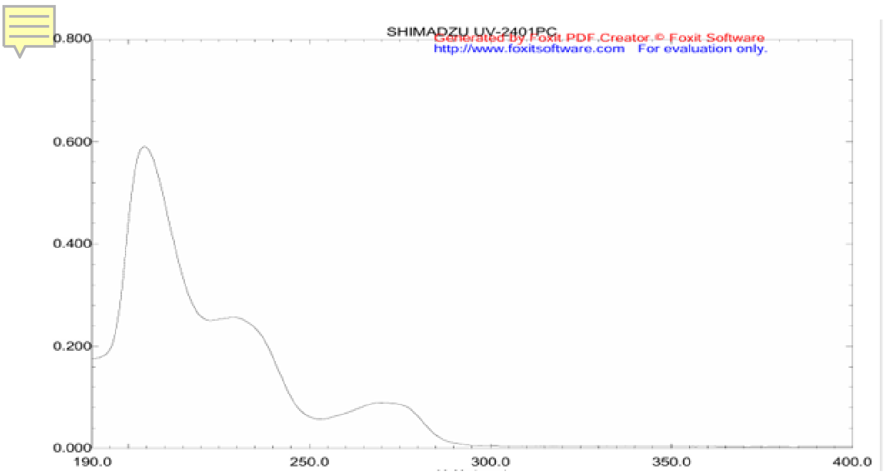

File Name: YPC-45 YPC-45

Created At: 16:35 25-11-21 Sample Concentration: 0.0074 mg/mL

Solvent: Methanol

Data: Raw

Measurement Mode: Abs. (Absorbance)

Scan Speed: Medium

Slit Width: 5.0

Sampling Interval: 0.5

No.	Wavelength (nm.)	Abs.
1	269.50	0.0896
2	229.00	0.2557
3	204.50	0.5906

Figure S22. IR spectrum of compound **2**.

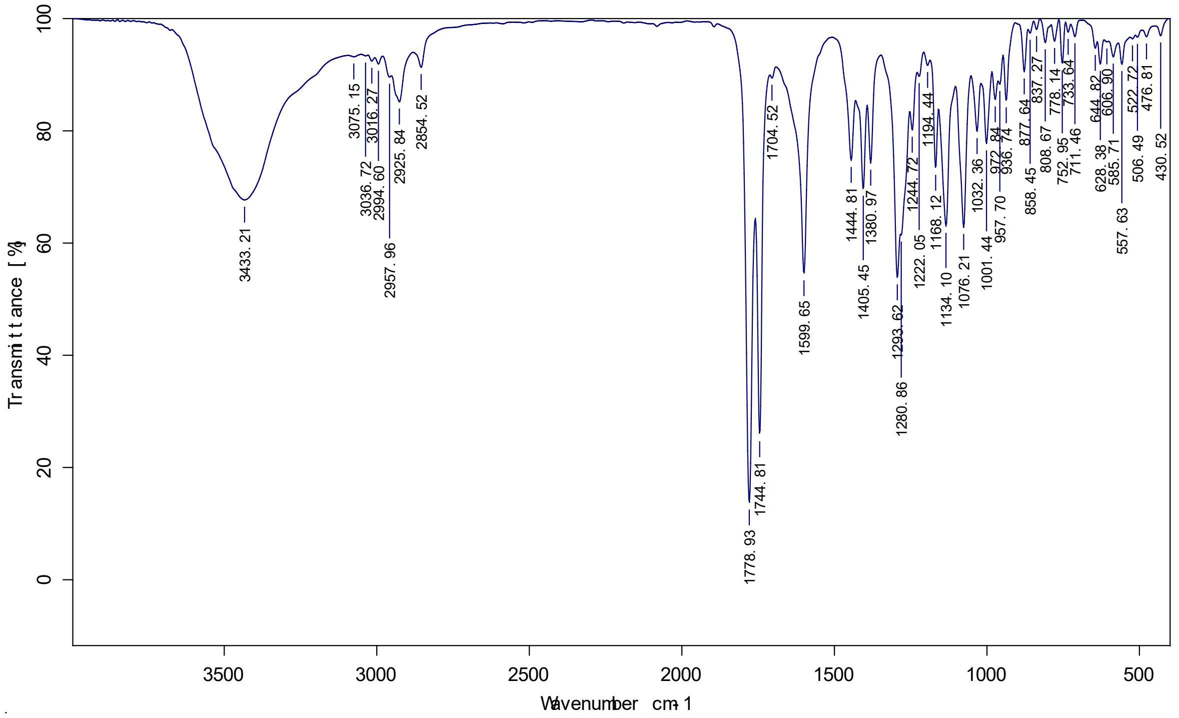

Sample Name: YPC-45	Resolution: 4	Beamsplitter Setting: KBr
Sample Form: KBr	Aperture Setting: 6 mm	Source Setting: MIR
Path of File: E:\data	Number of Background Scans: 16	Instrument Type: BRUKER VERTEX 70
Date of Measurement: 2025/11/24	Number of Sample Scans: 16	Soft Version: OPUS8.1

Figure S23. Optical rotation spectrum of compound **2**.

Rudolph Research Analytical

This sample was measured on an Autopol VI, Serial #91058
Manufactured by Rudolph Research Analytical, Hackettstown, NJ, USA.

Measurement Date : Monday, 24-NOV-2025

Set Temperature : 20.0

Time Delay : Disabled

Delay between Measurement : Disabled

n	Average	Std.Dev.	% RSD	Maximum	Minimum
5	7.01	0.71	10.12	7.79	6.49

S.No	Sample ID	Time	Result	Scale	OR °Arc	WLG.nm	Lg.mm	Conc.g/100ml	Temp.
1	YPC-45	12:58:49 PM	7.79	SR	0.006	589	100.00	0.077	20.0
2	YPC-45	12:58:55 PM	6.49	SR	0.005	589	100.00	0.077	20.0
3	YPC-45	12:59:02 PM	6.49	SR	0.005	589	100.00	0.077	20.0
4	YPC-45	12:59:08 PM	6.49	SR	0.005	589	100.00	0.077	20.0
5	YPC-45	12:59:15 PM	7.79	SR	0.006	589	100.00	0.077	20.0

Figure S24. CD spectrum of compound **2**.

YPC-45

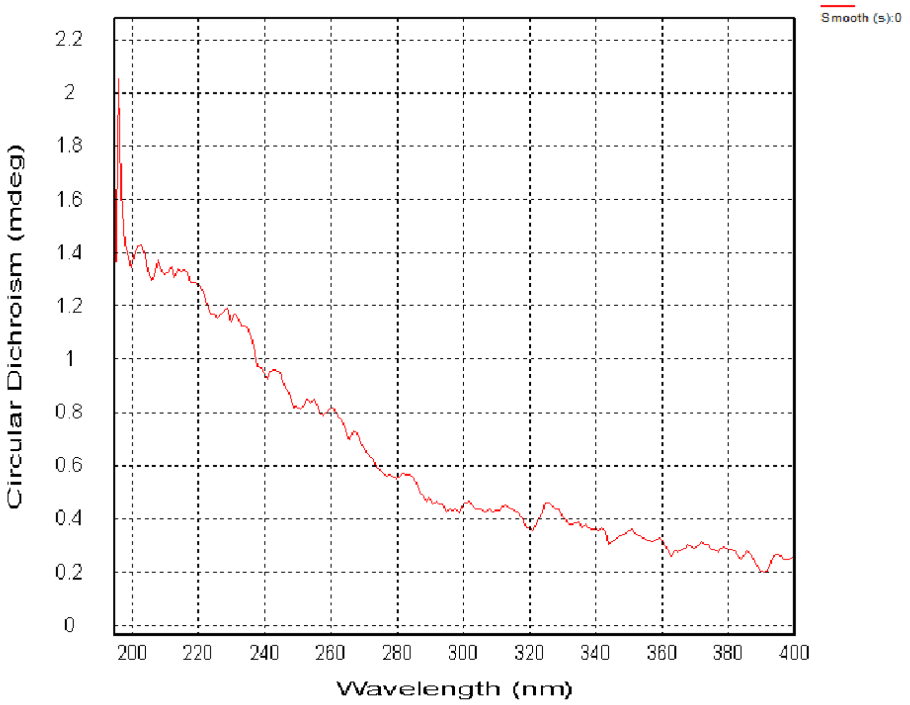

Figure S25. ^{1}H NMR spectrum of compound **3** in MeOD.

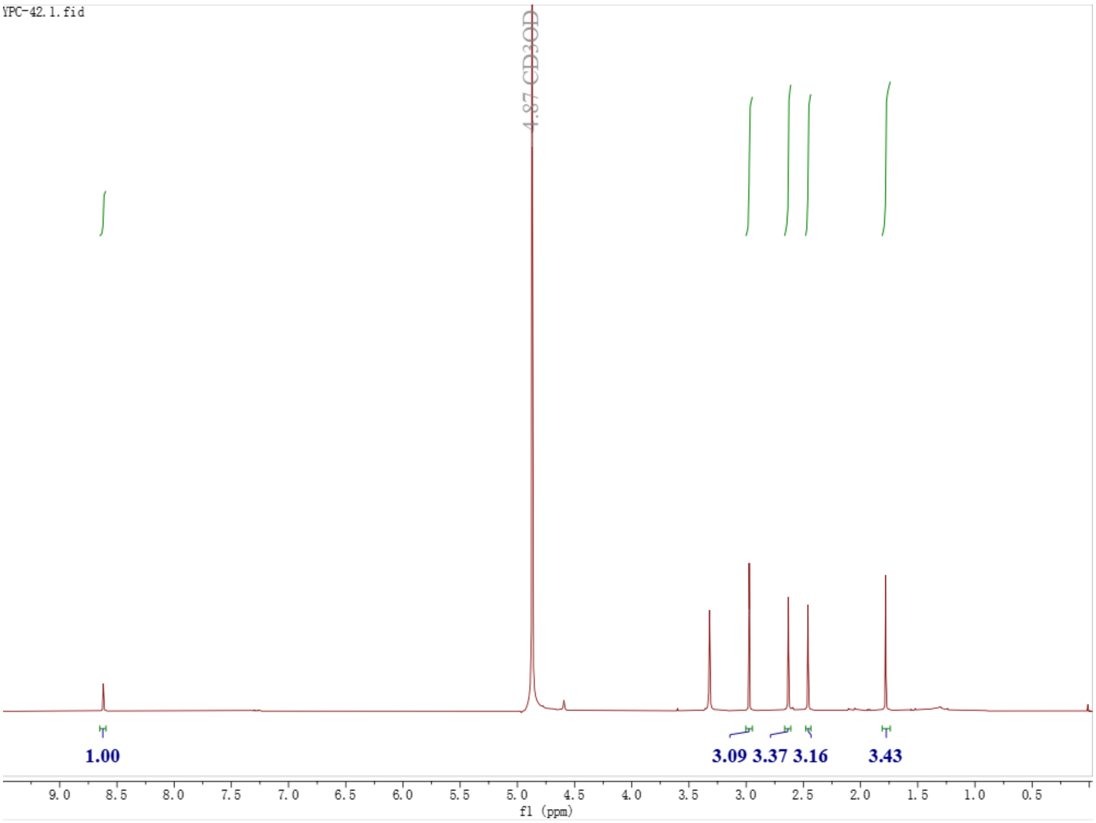

Figure S26. 13C NMR spectrum of compound **3** in MeOD.

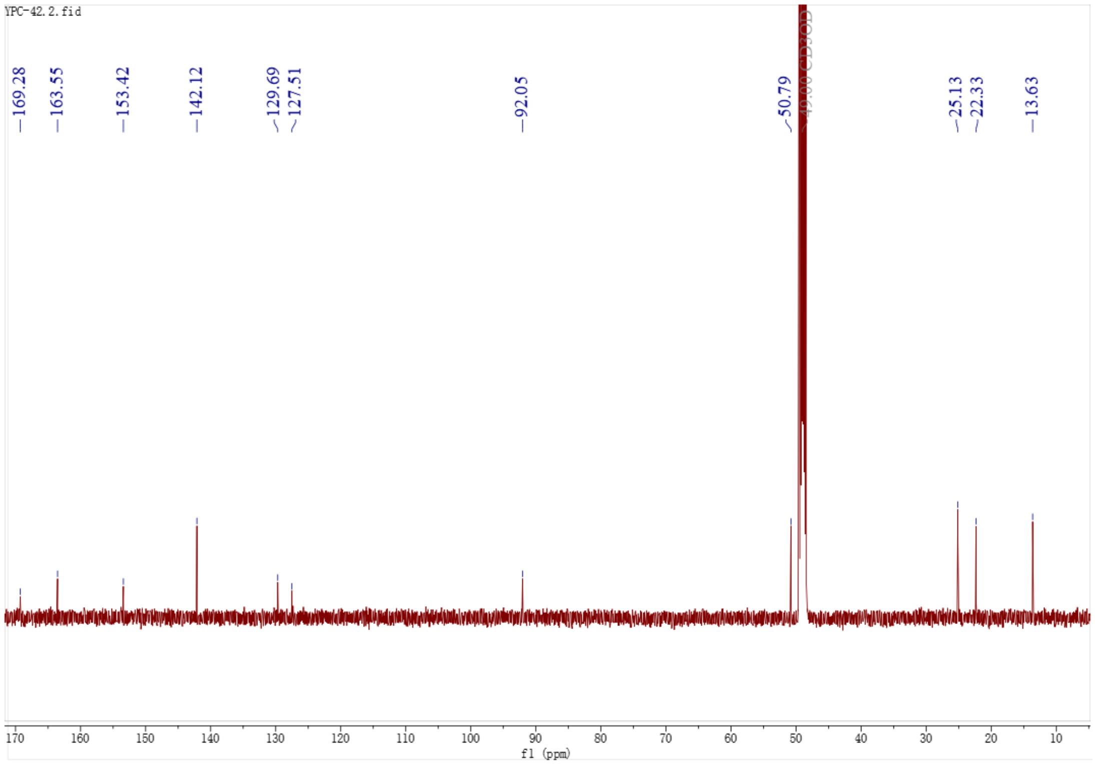

Figure S27. DEPT spectrum of compound **3** in MeOD.

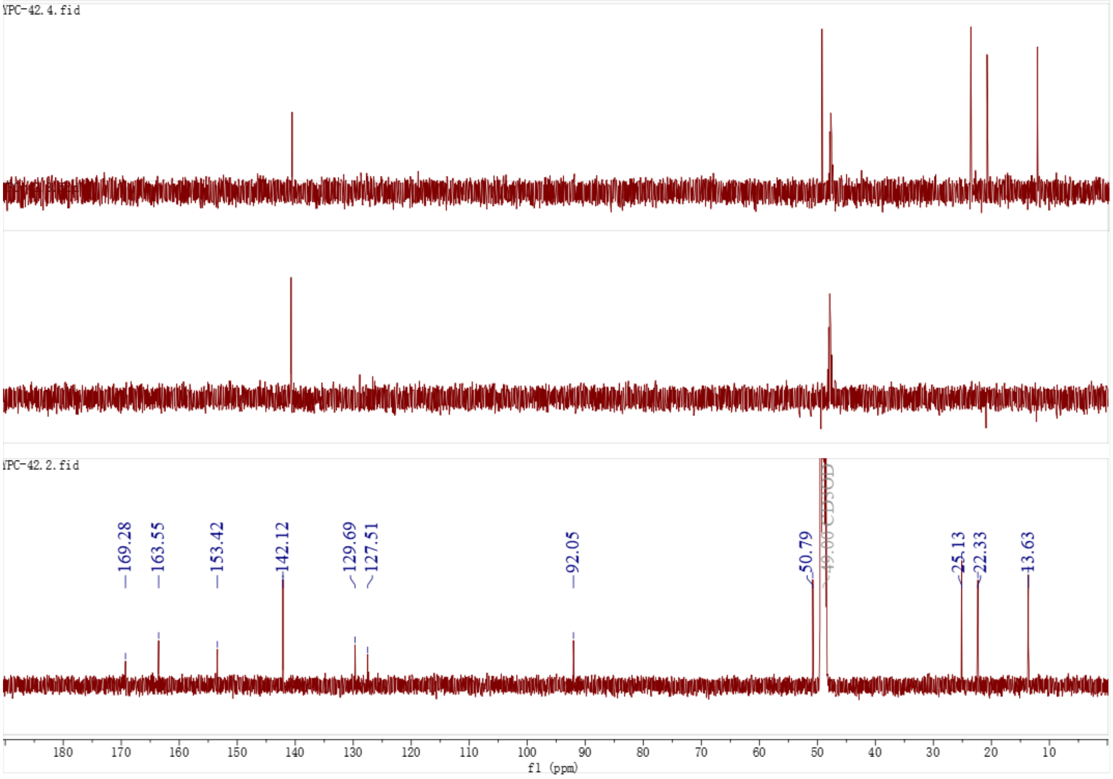

Figure S28. ^{1}H-^{1}H COSY spectrum of compound **3** in MeOD.

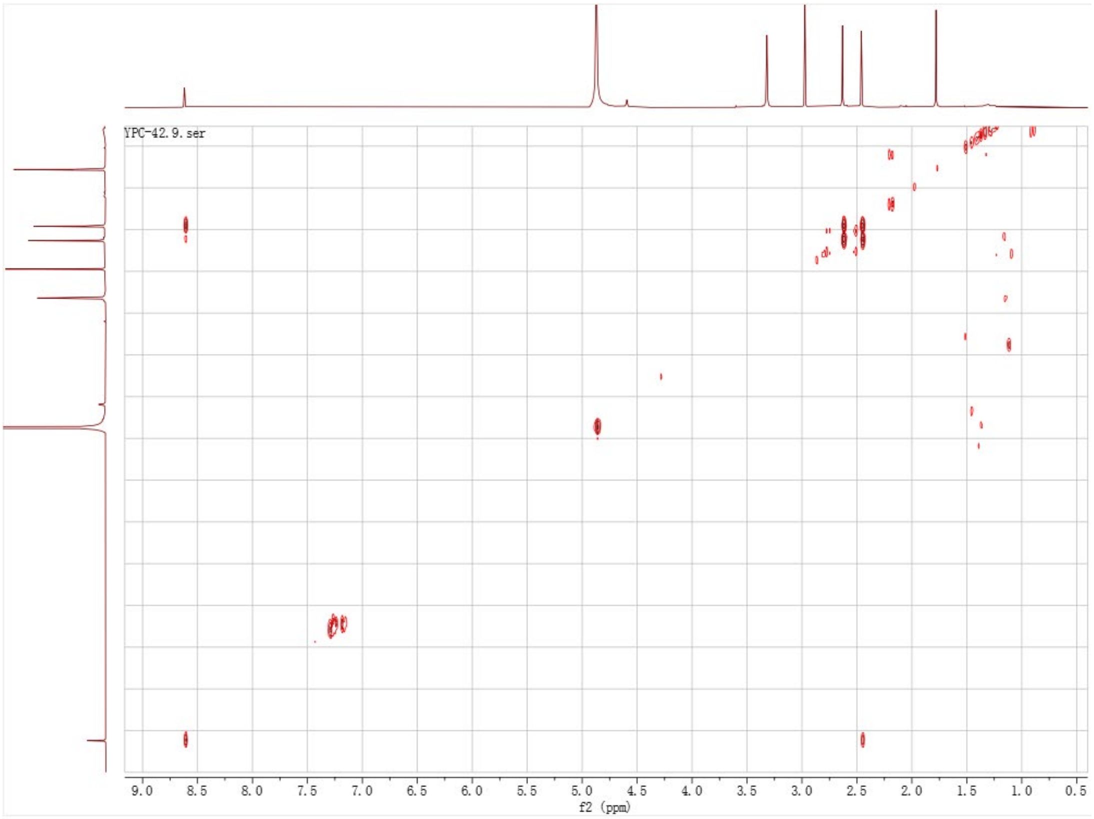

Figure S29. HSQC spectrum of compound **3** in MeOD.

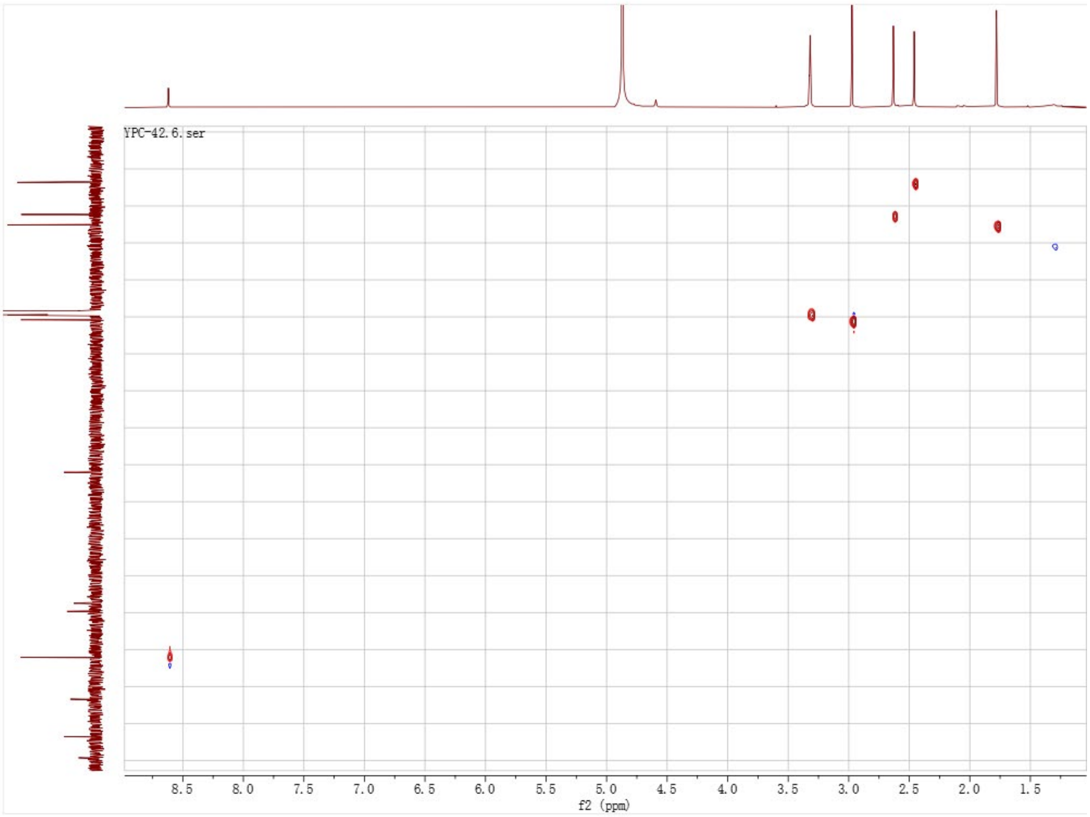

Figure S30. HMBC spectrum of compound **3** in MeOD.

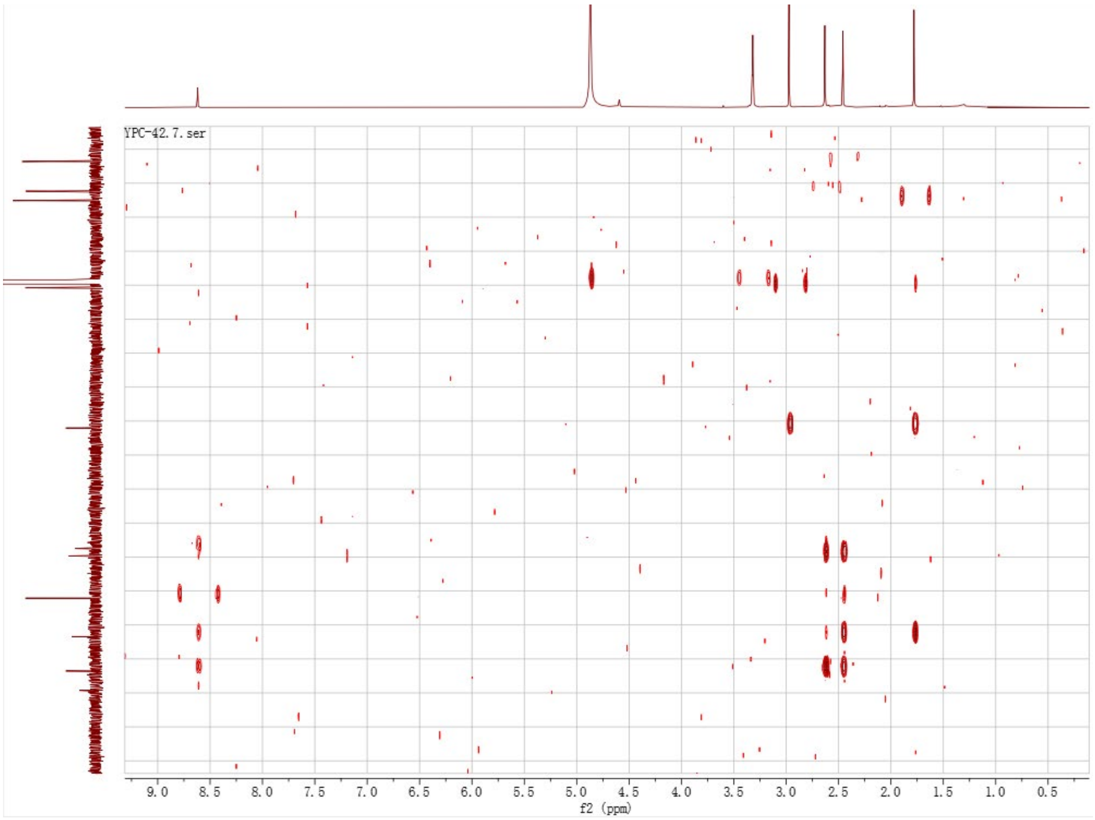

Figure S31. NOESY spectrum of compound **3** in MeOD.

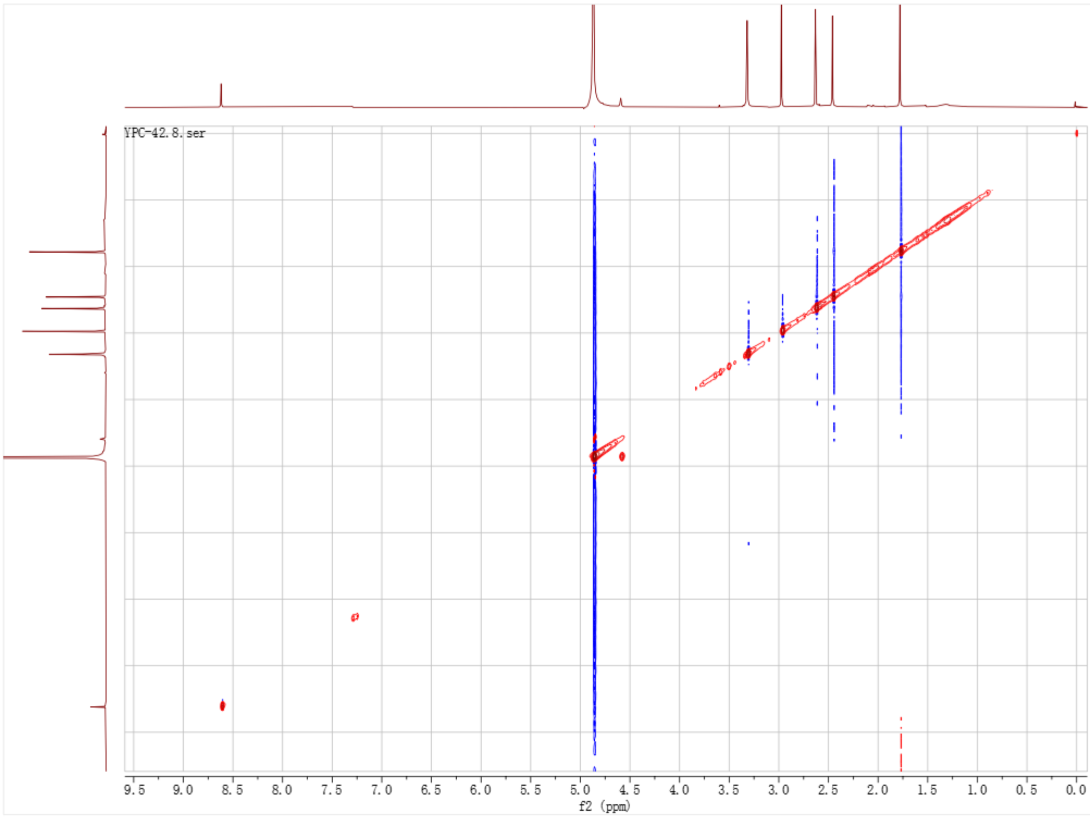

Figure S32. HR-TOF-ESI-MS spectrum of compound **3** in MeOD.

Qualitative Analysis Report

**Data Filename**	Ypc-42.d	**Sample Name**	Ypc-42
**Sample Type**	Sample	**Position**	Vial 14
**Instrument Name**	Agilent G6230 TOF MS	**User Name**	
**Acq Method**	ESI.m	**Acquired Time**	5/28/2025 4:07:11 PM
**IRM Calibration Status**	Success	**DA Method**	ESI.m
**Comment**			

**Sample Group**		**Info.**
**Acquisition SW Version**	6200 series TOF/6500 series Q-TOF B.05.01 (B5125.2)	

User Spectra

Fragmentor Voltage	Collision Energy	Ionization Mode
200	0	ESI

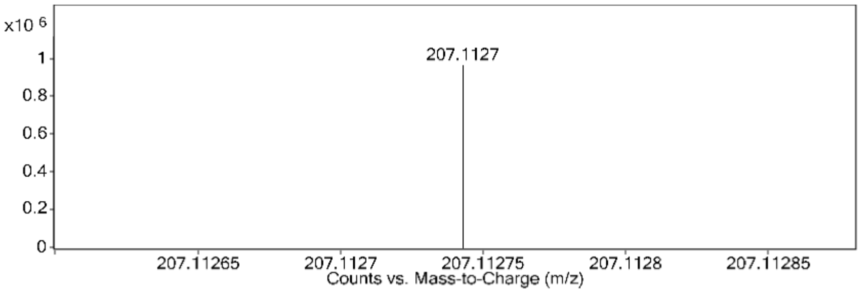

Peak List

m/z	z	Abund	Formula	Ion
121.0509	1	259201.38		
175.0862		128041.79		
176.0939	1	1075007.5		
177.0665		154420.47		
177.0939	1	153804.98		
207.1127	1	964925.81	C11 H15 N2 O2	M+
208.1155	1	115408.22	C11 H15 N2 O2	M+
274.2736	1	153139.22		
437.1938	1	101027.29		
922.0098	1	313237.19		

Formula Calculator Element Limits

Element	Min	Max
C	0	200
H	0	400
O	0	15
N	2	2

Formula Calculator Results

Formula	CalculatedMass	Mz	Diff.(mDa)	Diff. (ppm)	DBE
C11 H15 N2 O2	207.1134	207.1127	0.7	3.1	5.5

--- End Of Report ---

Figure S33. UV spectrum of compound **3**.

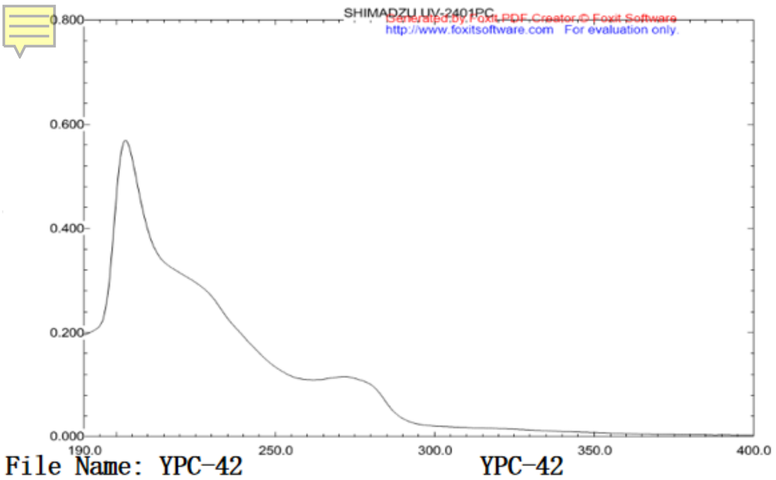

File Name: YPC-42

Created At: 17:13 25-09-09

YPC-42

Sample Concentration: 0.0097 mg/mL

Solvent: Methanol

Data: Raw

Measurement Mode: Abs. (Absorbance)

Scan Speed: Medium

Slit Width: 5.0

Sampling Interval: 0.5

No.	Wavelength (nm.)	Abs.
1	203.00	0.5697
2	217.50	0.3253
3	271.50	0.1160
4	304.50	0.0199

Figure S34. IR spectrum of compound **3**.

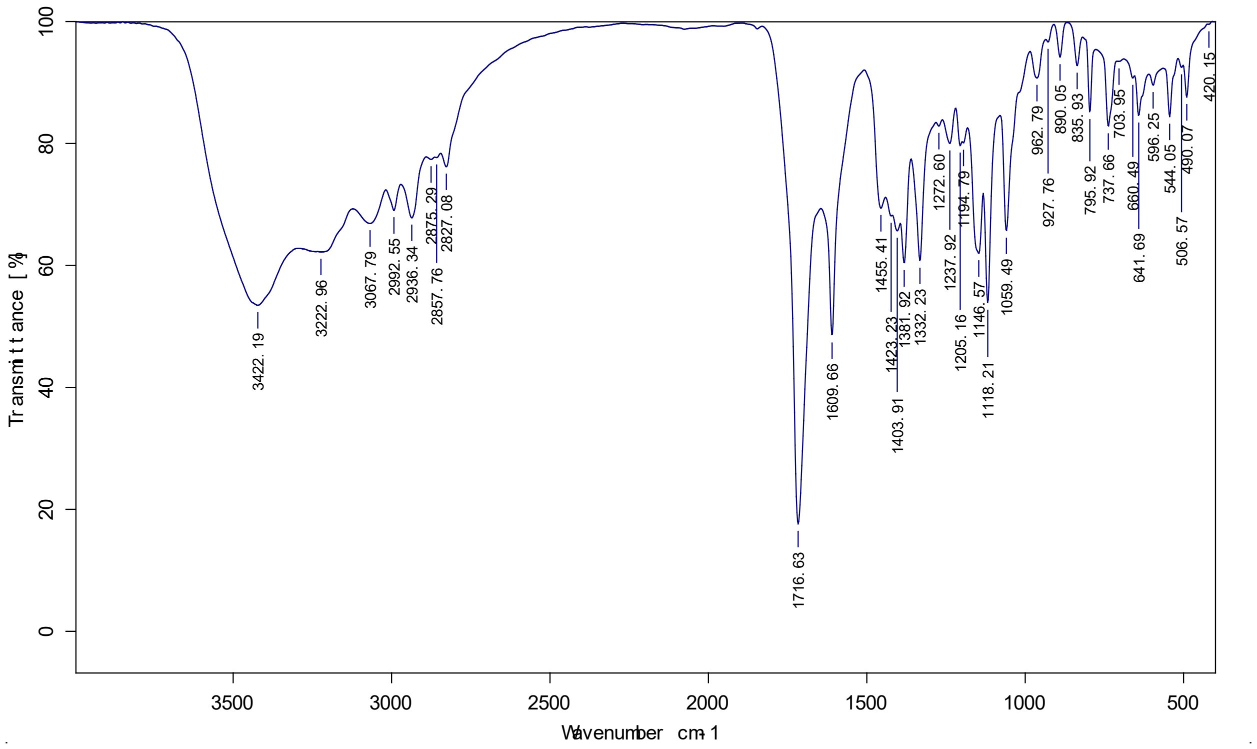

Sample Name: Ypc-42
Sample Form: KBr
Path of File: E:\data
Date of Measurement: 2025/9/12

Resolution: 4
Aperture Setting: 6 mm
Number of Background Scans: 16
Number of Sample Scans: 16

Beamsplitter Setting: KBr
Source Setting: MIR
Instrument Type: BRUKER VERTEX 70
Soft Version: OPUS8.1

Figure S35. Optical rotation spectrum of compound **3**.

Rudolph Research Analytical

This sample was measured on an Autopol VI, Serial #91058
Manufactured by Rudolph Research Analytical, Hackettstown, NJ, USA.

Measurement Date : Tuesday, 09-SEP-2025

Set Temperature : 25.0

Time Delay : Disabled

Delay between Measurement : Disabled

n	Average	Std.Dev.	% RSD	Maximum	Minimum
5	-4.60	0.89	-19.34	-3.00	-5.00

S.No	Sample ID	Time	Result	Scale	OR °Arc	WLG.nm	Lg.mm	Conc.g/100ml	Temp.
1	YPC-42	02:23:53 PM	-3.00	SR	-0.003	589	100.00	0.100	25.0
2	YPC-42	02:24:00 PM	-5.00	SR	-0.005	589	100.00	0.100	25.0
3	YPC-42	02:24:06 PM	-5.00	SR	-0.005	589	100.00	0.100	25.0
4	YPC-42	02:24:13 PM	-5.00	SR	-0.005	589	100.00	0.100	25.0
5	YPC-42	02:24:20 PM	-5.00	SR	-0.005	589	100.00	0.100	25.0

Figure S36. CD spectrum of compound **3**.

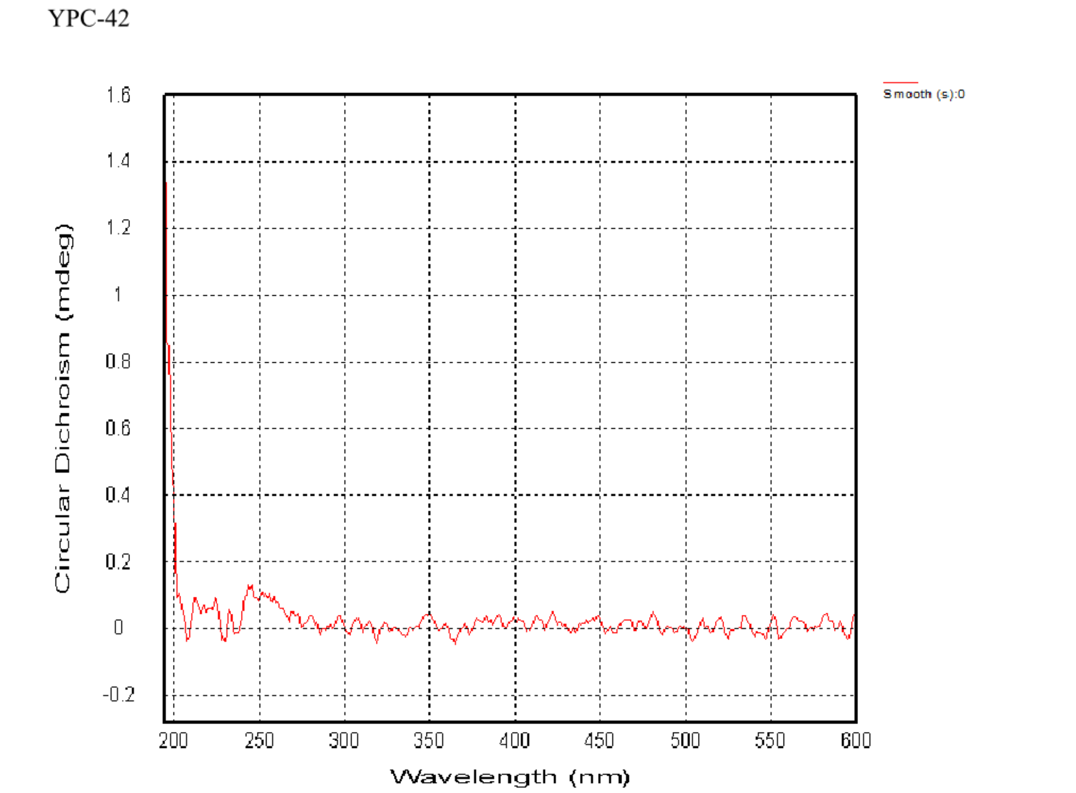

Figure S37. Fungal inhibition experiment of compounds **1-5**.

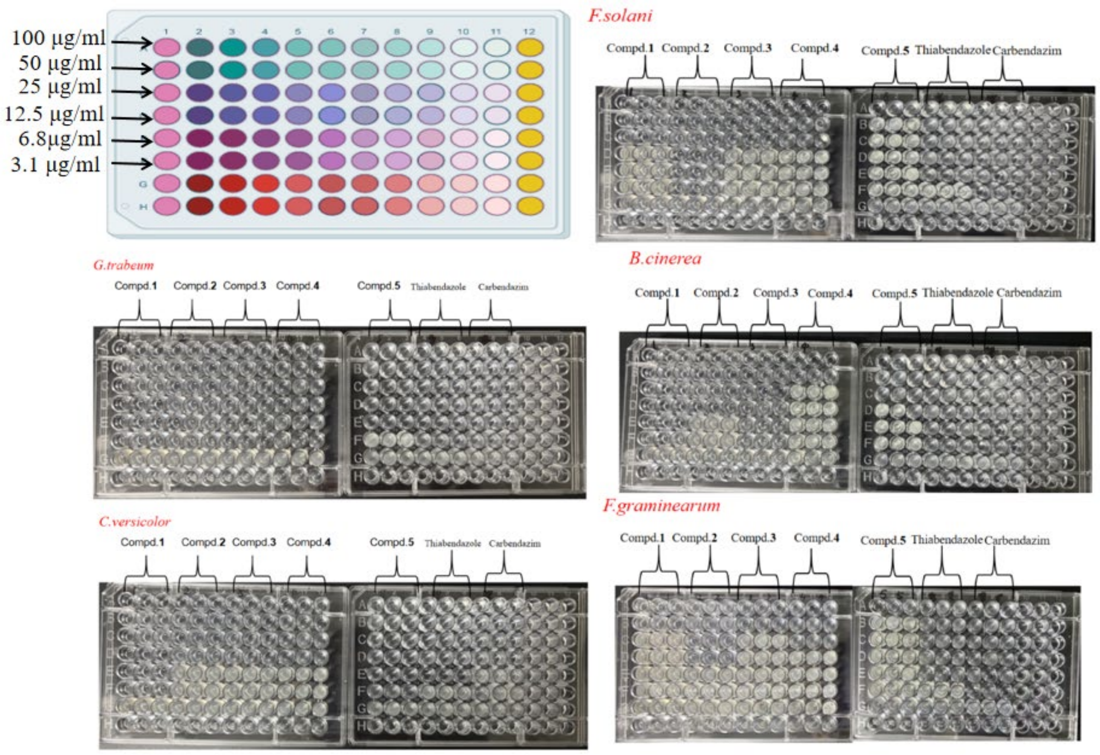

Supplement: Supplementary file 1 [file jof-12-00296-s001.zip › jof-4210969-supplementary.pdf]
